# A Unified Method for Detecting Secondary Trait Associations with Rare Variants: Application to Sequence Data

**DOI:** 10.1371/journal.pgen.1003075

**Published:** 2012-11-15

**Authors:** Dajiang J. Liu, Suzanne M. Leal

**Affiliations:** 1 Department of Biostatistics, Center of Statistical Genetics, University of Michigan, Ann Arbor, Michigan, United States of America; 2 Department of Molecular and Human Genetics, Baylor College of Medicine, Houston, Texas, United States of America; Georgia Institute of Technology, United States of America

## Abstract

Next-generation sequencing has made possible the detection of rare variant (RV) associations with quantitative traits (QT). Due to high sequencing cost, many studies can only sequence a modest number of selected samples with extreme QT. Therefore association testing in individual studies can be underpowered. Besides the primary trait, many clinically important secondary traits are often measured. It is highly beneficial if multiple studies can be jointly analyzed for detecting associations with commonly measured traits. However, analyzing secondary traits in selected samples can be biased if sample ascertainment is not properly modeled. Some methods exist for analyzing secondary traits in selected samples, where some burden tests can be implemented. However p-values can only be evaluated analytically via asymptotic approximations, which may not be accurate. Additionally, potentially more powerful sequence kernel association tests, variable selection-based methods, and burden tests that require permutations cannot be incorporated. To overcome these limitations, we developed a unified method for analyzing secondary trait associations with RVs (STAR) in selected samples, incorporating all RV tests. Statistical significance can be evaluated either through permutations or analytically. STAR makes it possible to apply more powerful RV tests to analyze secondary trait associations. It also enables jointly analyzing multiple cohorts ascertained under different study designs, which greatly boosts power. The performance of STAR and commonly used RV association tests were comprehensively evaluated using simulation studies. STAR was also implemented to analyze a dataset from the SardiNIA project where samples with extreme low-density lipoprotein levels were sequenced. A significant association between *LDLR* and systolic blood pressure was identified, which is supported by pharmacogenetic studies. In summary, for sequencing studies, STAR is an important tool for detecting secondary-trait RV associations.

## Author Summary

Next-generation sequencing has greatly expanded our ability to identify missing heritability due to rare variants. In order to increase the power to detect associations, one desirable study design is to combine samples from multiple cohorts for mapping commonly measured traits. However, many current studies sequence selected samples (e.g. samples with extreme QT), which can bias the analysis of secondary traits, unless the sampling ascertainment mechanisms are properly adjusted. We developed a unified method for detecting secondary trait associations with rare variants (STAR) in selected and random samples, which can flexibly incorporate all rare variant association tests and allow joint analysis of multiple cohorts ascertained under different study designs. We demonstrate via simulations that STAR greatly boosts the power for detecting secondary trait associations. As an application of STAR, a dataset from the SardiNIA project was analyzed, where DNA samples from well-phenotyped individuals with extreme low-density lipoprotein levels were sequenced. *LDLR* was identified to be significantly associated with systolic blood pressure, which is supported by a previous pharmacogenetics study. In conclusion, STAR is an important tool for sequence-based association studies.

## Introduction

Next-generation sequencing has already revolutionized the study of complex traits, and made possible the detection of rare variant associations. Many sequence based association studies are currently being performed, some of which have already lead to the discovery of associations with clinically important traits, such as lipids levels [1], age related macular degeneration [2], inflammatory bowel disease [3], blood pressure [4], body mass index [5], etc. In particular, there is increasing interest to detect associations with quantitative traits (QT). It has been suggested that complex traits can be due to multiple variants with small effects, and are quantitative in nature [6], [7]. Mapping multiple quantitative traits may help elucidate the etiology of complex traits, reducing sample heterogeneity [8], dissecting gene pleiotropy and refine the definition of complex diseases [7], [9], [10]. For example, recent studies of type 2 diabetes have been focused on multiple related QTs, such as fasting glucose levels [11], insulin resistance levels [11], and c-reactive proteins [12]. Many quantitative traits are usually measured in different studies as secondary outcomes. It is of great interest to combine multiple studies for detecting associations with commonly measured primary or secondary traits. For example, the National Heart Lung and Blood Institute-Exome Sequencing Project (NHLBI-ESP) is studying a variety of different phenotypes and employed both extreme and random sampling. In order to improve power, all samples with the phenotype of interest measured are jointly analyzed. Specifically, the association analysis of low density lipoprotein cholesterol (LDL) levels is performed by combining several studies which include a well-phenotyped random population cohort, selected samples with extreme LDL levels as well as individuals with extreme body mass index (BMI).

Many methods have been developed for detecting rare variant associations [13]–[19]. These methods are all based upon aggregating multiple rare variants across a genetic region, which is usually a gene. Compared to analyzing each variant individually, these region based tests can be more powerful. However, rare variants that are involved in complex trait etiologies usually only have moderate effect sizes, and their aggregated frequencies across a genetic region can still be low. It is therefore necessary to sequence and analyze a large number of samples in order to have adequate power to detect associations. Although next generation sequencing is much more cost effective than Sanger sequencing, it is still expensive to generate high read depth data for large numbers of samples. Given cost constraints, in order to improve power, many studies sequence samples with extreme QT rather than the entire cohort. The selective sampling study design produces challenges for analyzing secondary traits. Without properly accounting for the sample ascertainment mechanisms, type I errors for detecting secondary trait associations may be inflated [20], [21]. This is because due to the correlations between the primary and secondary traits, mean values for the secondary traits will be different between individuals with primary trait from opposite extreme tails. Additionally if the primary trait is associated with a gene region, the cumulative variant frequencies will also be different. Therefore spurious association can be created by ascertainment. The bias for the naïve analysis of secondary trait is demonstrated both theoretically and empirically in this article.

Several methods exist for detecting secondary trait associations in selected samples. For example, a retrospective likelihood method was developed for mapping secondary phenotypes using regression models (SPREG) in case control studies [20]. It was subsequently extended in an empirical Bayesian framework [22], which utilizes genotype information from cases for rare diseases and can be more powerful than the retrospective likelihood method. However, both methods are not directly applicable to the studies where the primary trait is quantitative and extreme sampling is implemented. We previously developed a method for detecting multiple (secondary) trait associations (MTA) in selected samples, which jointly models multiple phenotype associations and sampling ascertainment status [21]. MTA can be used to analyze data from studies with known sampling mechanisms, e.g. case control, and extreme sampling designs. It incorporates several rare variant association tests, whose statistical significance can be evaluated analytically e.g. the combined multivariate and collapsing (CMC) [16], and Gene- or Region-based Analysis of Variants of Intermediate and Low frequency (GRANVIL) [18]. Weighted sum statistics [14], [23] can also be incorporated, if the weights that are assigned to each variant site are not dependent on the trait. One major advantage of using MTA is that cohorts ascertained under different sampling schemes can be combined for detecting associations with commonly measured traits. These studies can be targeted at the same or different primary traits. By combining data from different studies, much larger sample sizes can be analyzed and the power to detect associations can be greatly improved [21].

However none of these methods for detecting secondary associations incorporate sequence kernel association test (SKAT), a powerful variance component score test based method. This method can be more powerful when causal variants have bidirectional effects and/or a large proportion of the variants within gene region are non-causal.

Standard permutation algorithms cannot be applied to obtain empirical p-value. This is because when the primary and secondary traits are correlated and the genetic region is associated with the primary trait, neither the secondary trait residuals nor the locus genotypes are interchangeable under the null hypothesis. Therefore, the statistical significance can only be evaluated via asymptotic approximations, which has several notable limitations: 1.) Due to the low frequency of rare variants, asymptotic approximation for some tests may be violated, which can lead to either inflated type I error or loss of power. 2.) For some rare variant association methods, the analytical distribution for the test statistics is unknown and therefore the statistical significance has to be evaluated empirically. These rare variant tests that require evaluating p-values via permutation are often more powerful than the methods implemented in MTA, e.g. CMC or GRANVIL. It is therefore desirable that these tests can be applied to analyze secondary traits.

To overcome the limitations of existing methods, a unified model was developed to detect secondary trait associations using selected samples. In the samples with extreme primary quantitative traits, through re-parameterizing the likelihood functions, interchangeable residuals for the secondary traits can be obtained under the null hypothesis. The residuals are approximately independent, and normally distributed. We proved theoretically that the analysis of secondary trait associations can be equivalently implemented by analyzing the correlation between the secondary trait residuals and the gene/genetic region. Therefore, any rare variant association test that can analyze QT in random population based studies can be incorporated in STAR. In addition, multiple cohorts can be jointly analyzed through conventional mega-analysis methods that use individual participant data or meta-analysis methods that use summary level statistics.

A variety of popular rare variant tests have been implemented in the STAR framework and the power to detect secondary trait associations was evaluated. Specifically, we considered the weighted sum statistic (WSS) [14], [23], sequence kernel association tests (SKAT) [17], and variable threshold test (VT) [24]. Additionally the kernel based adaptive cluster test (KBAC) [15], which was originally developed for analyzing binary disorders, was extended to analyze quantitative traits and incorporated in STAR for detecting associations with secondary phenotypes (Text S1).

The performances for these methods were compared using extensive simulation studies. Genetic data were simulated under a realistic population genetic model as described by Kryukov et al [25], which incorporates both demographic change and purifying selections. Phenotypes were simulated based upon parameters estimated from clinically important complex traits. It is demonstrated that under a broad variety of phenotype models, the power for detecting secondary trait associations can be greatly improved through the use of more powerful rare variant tests that are incorporated in STAR. There does not exist a method that is consistently the most powerful, and the power difference between top performing methods is generally small. When the effects of causal variants are unidirectional, the VT test outperforms other methods in most scenarios. When there are variants with effects in opposite directions or only a small proportion of the variants are causal, SKAT can be more powerful than alternative methods in many settings.

The STAR method was also used to analyze a published sequence dataset from the SardiNIA project [1], where nine genes were sequenced in 256 individuals with extreme LDL levels (individuals taking lipid-lowering therapies were not considered for the analysis). In the original article by Sanna et al [1], the authors focused on detecting associations with the primary trait LDL, and did not consider analyzing other metabolic and lipids traits. In this article, the analysis was extended to detect associations with other clinically important traits, which include high density lipoprotein cholesterol (HDL), total cholesterol level (TCL), triglyceride (TG), insulin levels (INSULIN), BMI, systolic and diastolic blood pressure (SysBP and DiasBP). One association was identified between *LDLR* and SysBP, which is statistically significant after applying a Bonferroni correction for testing multiple genes and traits. This association has strong biological support from pharmacogenetics studies [26]. These findings provide new insight on the etiology for the *LDLR* gene, and established the importance of our method in sequence based association studies.

An R-package, STARSEQ which implements the STAR method is available through the Comprehensive R Archive Network (CRAN) at http://cran.r-project.org/web/packages/STARSEQ/. Additional companion softwares are deposited at http://code.google.com/p/starseq/.

## Materials and Methods

STAR model can be used with any rare variant test to detect associations with secondary traits in studies that use extreme sampling. The multi-site genotype for individual 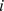 is denoted by a vector 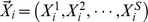, where 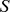 is the total number of variant sites that are jointly analyzed. The set of variants can be determined by variant frequency threshold (either fixed or variable), or functional annotations (e.g. non-synonymous variants) etc. Each entry in the vector 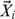 can be coded by the number of minor alleles, e.g. 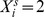 if the genotype is homozygous for the minor allele. The primary QT is denoted by 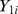, while the secondary trait under consideration is denoted by 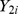. In vector (or matrix) notations, the primary traits, secondary traits and genotypes for the entire sample are respectively denoted by 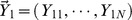, 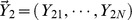 and 
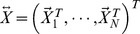
.

### Ascertainment Corrected Likelihood Model under the Null Hypothesis

Under the null hypothesis of no gene/secondary trait associations, following the MTA framework, a multivariate generalized linear model can be implemented to estimate nuisance parameters [21]. The link functions for the mean parameters of the two traits satisfy
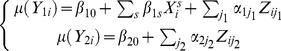
(1)In the above model, 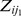 and 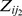 are covariates, such as age or sex.

The residual terms for the secondary traits, i.e. 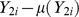 are correlated with the primary trait, and not interchangeable under the null hypothesis, i.e.
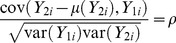
(2)It was previously shown via simulations that naïve inferences for secondary trait associations, which ignore sample ascertainment mechanisms, can be biased [20], [21]. It can also be proved theoretically that due to extreme sampling on the primary trait, spurious associations can be created between the gene locus and secondary trait (Text S2, Figures S1 and S2). Without adjusting for the sample ascertainment mechanisms, the biases in the secondary trait effects will increase linearly with respect to the trait residual correlation 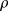 and approximately linearly with respect to the primary trait effects when the magnitude of primary trait effects is small. Using this theoretical framework, we also evaluated some standard adjustment methods. e.g. 1.) Separately analyzing individuals with high and low extreme primary traits, and then combining the results via meta-analysis. or 2.) Incorporating an indicator to denote whether an individual has a high or low extreme primary trait as a covariate, and perform linear regression analysis using the entire sample. We proved theoretically that these methods will not eliminate the bias in the association analysis of secondary traits, and type I error will still be inflated after the adjustment.

In order to obtain unbiased results, sampling schemes have to be properly modeled. Ascertainment corrected likelihood can be used, which jointly models sample ascertainment status 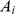 and genotype/phenotype association, i.e.
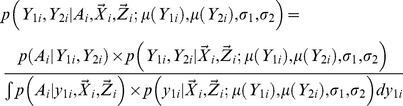
(3)The likelihood model can be used for both trait dependent sampling and population based random sampling. We showed analytically that the secondary trait effects can be consistently inferred under the ascertainment corrected likelihood model. Details for the likelihood specification can be found in (Text S3).

### Re-Parameterization of the Likelihood Function

The likelihood function in equation (1) needs to be re-parameterized in order to facilitate deriving the SKAT statistics and performing permutations. It is clear that
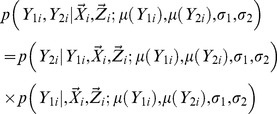
(4)The conditional probability 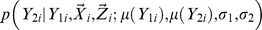 satisfies
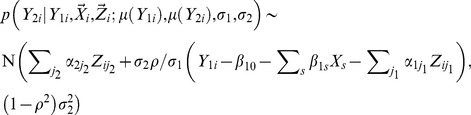
(5)Instead of estimating the variance and correlation coefficients for 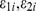, the following parameters are estimated, i.e. 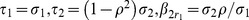. As is shown in (Text S3), the Jacobian for the re-parameterization, i.e. 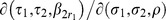 is non-degenerate and the re-parameterization is one to one and invertible. Therefore, an equivalent mean model can be fitted under the null hypothesis, i.e.
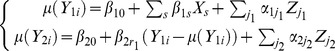
(6)Practical issues for fitting the model are discussed in (Text S4).

In this model, the residual errors 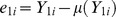 and 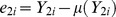 for the primary and secondary traits are uncorrelated. In particular, the residual errors 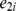 after re-parameterization are interchangeable under the null hypothesis.

### Score Statistics for Burden Tests and Variance Component Score Test

Burden tests, such as CMC and WSS, aggregate multiple rare variants across a genetic region and analyze them jointly. The following model can be used to obtain score statistics for a burden test:
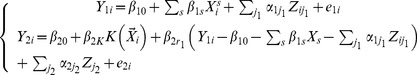
(7)In formula (7), 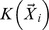 is the genotype coding for the locus multi-site genotypes. Examples include the weighted sum coding [14], i.e. 
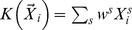
, where each variant site is assigned a weight and the weighted genotypes are aggregated. For some rare variant association tests such as KBAC [15], the genotype coding can also depend on the QT, i.e. 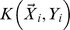. Formula 7 can be used for detecting single variant associations as well, where 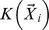 is the coding for single variant genotype.

Score tests can be formally constructed from the joint likelihood for testing the null hypothesis of no gene/secondary trait associations, i.e. 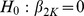. If the samples are ascertained based upon only the primary trait, score tests can be equivalently constructed from the conditional likelihood, i.e. 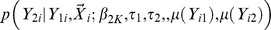. This is because the joint likelihood can be factorized, i.e.
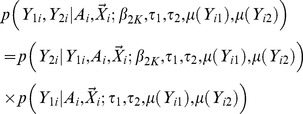
(8)When the samples are ascertained based upon the primary trait, the distribution of 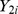 conditional on 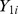 is independent of the ascertainment status 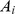, i.e.
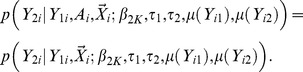
In addition, the term 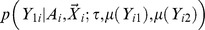 does not contain the parameter of interest 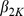. The score function thus takes the form
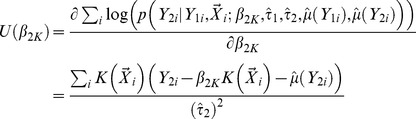
(9)where 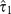, 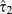, 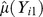 and 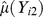 are maximum likelihood estimates under the null hypothesis.

It is clear from formula (9) that 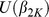 is proportional to the covariance between the secondary trait residuals and the locus genotype coding. Given that 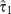, 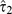, 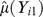 and 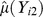 are consistent estimators under the null hypothesis, by Slutsky's theorem, the residuals for the secondary trait i.e. 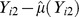 are approximately normally distributed and interchangeable under the null hypothesis. Therefore, the analysis of rare variant secondary trait associations can be implemented by analyzing the correlation between the corrected residuals and the locus genotype coding. Standard permutation algorithms can be implemented by shuffling the residuals under the null hypothesis. In our STARSEQ package, we also provide flexible tools for calculating the adjusted secondary trait residuals, which can be analyzed by any user specified rare variant association test.

Using similar ideas, we show in (Text S5), that the extended SKAT statistic in STAR has the form
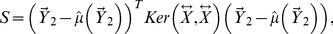
(10)where 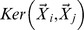 is the kernel function used to compare two multi-site genotypes 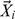 and 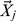, and 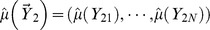 is the estimated mean secondary trait value under the null model. P-values for the extended SKAT method can be obtained either analytically or via permutation.

### Extensions of KBAC Test to Analyze Quantitative Trait Associations

The KBAC test was previously developed for the analysis of binary trait associations [15]. It is extended to analyze rare variant QT associations in studies using randomly ascertained samples or samples with extreme traits. The extended KBAC method has also been incorporated in STAR for analyzing secondary trait associations. The details for the extensions are given in (Text S1).

### Evaluation of Type I Errors and Power

Type I error and power were evaluated for the following rare variant association tests that were extended in STAR, i.e. CMC, KBAC, WSS, SKAT and VT. Genetic data were generated according to a four parameter demographic model for Europeans [25], [27]. In addition, purifying selection is also modeled, which influences the variant site frequency spectrum. Among the variants with selection coefficients >10^−4^, 50% are randomly chosen to be causal for the primary trait, and another 50% of the variants are independently chosen to be causal for the secondary trait. The set of causal variants for the primary trait are denoted by 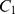 and that for the secondary trait are denoted by 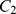. Variants belonging to the intersection of 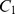 and 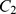 modulate both the primary and secondary phenotypes. QTs were simulated according to the following bivariate normal distribution:
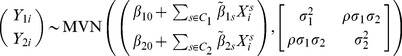
(11)The magnitudes of the causal variant effects are assumed to be inversely correlated with the minor allele frequencies (MAF) 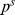, i.e.
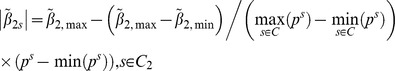
For a special case when 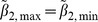, the magnitude for the effects of all rare causal variants is constant, i.e. 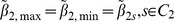. In the simulations, we considered models where 1.) 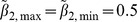
2.) 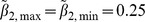 and 3.) 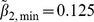 and 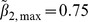. For each set of parameter values of 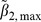 and 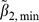, we evaluated the power for different rare variant association tests when 1.) all causal variants have effects in the same direction or 2.) 80% of the variants increase the mean secondary trait value while the remaining 20% decrease the mean secondary trait value. In the simulations, the primary and secondary traits are assumed to be positively (or negatively) correlated with coefficients 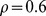 (or 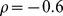). For the evaluation of type I errors, datasets were simulated with 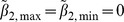.

Data for selective sampling studies are simulated, where for each dataset, a total of 5,000 individuals with extreme primary trait values are selected from a cohort of 100,000 individuals. Two-sided alternative hypothesis was tested for each method. Although p-values for CMC and SKAT can be obtained analytically, they can either be conservative or anti-conservative [23]. In order to calibrate the distribution of p-values, we evaluated the statistical significance of all methods empirically using 5,000 permutations. The power and type-I errors for each method were obtained using 10,000 replicates for a significance level of 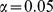. As a comparison to STAR, type I error for linear regression analysis was also evaluated, where sample ascertainment mechanisms were ignored.

### Application of STAR to Meta-Analysis

In order to illustrate the application of STAR for combining multiple cohorts, a meta-analysis of three studies was simulated. The primary trait for each study is different and a common secondary trait is measured for all studies. In the first study, the gene region is associated with the primary trait, and causal variants have an effect of −0.5. The correlation between the primary and secondary traits is 0.6. In the second study, the primary trait is associated with the gene region, and causal variants have an effect of 0.25. The primary and secondary traits are correlated with coefficient 0.4. In the third study, the gene region is not associated with the primary trait, and the correlation between the primary and secondary traits is −0.2. In each study, a different pool of 50,000 samples was simulated and 2,500 individuals with extreme primary trait were selected and analyzed for association. P-values for all rare variant tests in each study were obtained based upon 5,000 permutations. Meta-analysis is performed by combining Z-score statistics, which are transformed from p-values and weighted by the square root of the sample sizes in each study [28].

In order to evaluate type I errors, data were simulated under the assumption that the secondary trait effects for all variants were 0. The empirical distribution of p-values was obtained using 10,000 replicates. For evaluating power, two scenarios were considered, i.e. (A) causal variants have an unidirectional effect of 0.5; (B) causal variants have bidirectional effects, where 80% of the causal variants have effect 0.5 and the other 20% of the causal variants have effect −0.5.The power for analyzing each individual study and meta-analysis was evaluated using 10,000 replicates under a significance level of α = 0.05.

### The Analysis of the SardiNIA Sequence Dataset

Association analyses were performed for the nine genes that were sequenced from the SardiNIA project [1]. First, coding regions of four genes, *APOB*, *B3GA4*, *LDLR* and *PCSK9* were tested for associations with the eight metabolic QTs. The genes *APOC1*, *APOC2*, *APOE*, *B4GA4* contain no variants with MAF<1%, and *SORT1* contains only 1 rare variant site. Gene-based association analysis was not performed for these five genes. Among the 256 individuals, 33 were taking blood pressure (BP) lowering medications; and their BP levels were adjusted by adding 10 mm Hg to their SysBP and 5 mm Hg to DiasBP levels [29]. Following the same strategy as the initial LDL analysis [1], residuals for each trait were obtained and quantile-normalized after adjusting for age, age×age and sex in the entire SardiNIA cohort. The normalized residuals of the 256 samples were analyzed for associations with the four genes, i.e. *APOB*, *B3GA4*, *LDLR* and *PCSK9*. The five rare variant association tests incorporated in STAR were used to analyze the data. In addition to the secondary traits, the associations with the primary trait (i.e. LDL levels) were also analyzed. For the rare variant tests that use fixed MAF thresholds (i.e. CMC, WSS, KBAC and SKAT), variants with MAF<1% were analyzed. For VT test, variants with MAF<5% were used in the analysis. The secondary traits were also analyzed using standard linear regression that ignores the ascertainment mechanism, as a comparison to the analysis using the STAR method.

## Results

### Evaluation of Type I Error

**Figure 1:**
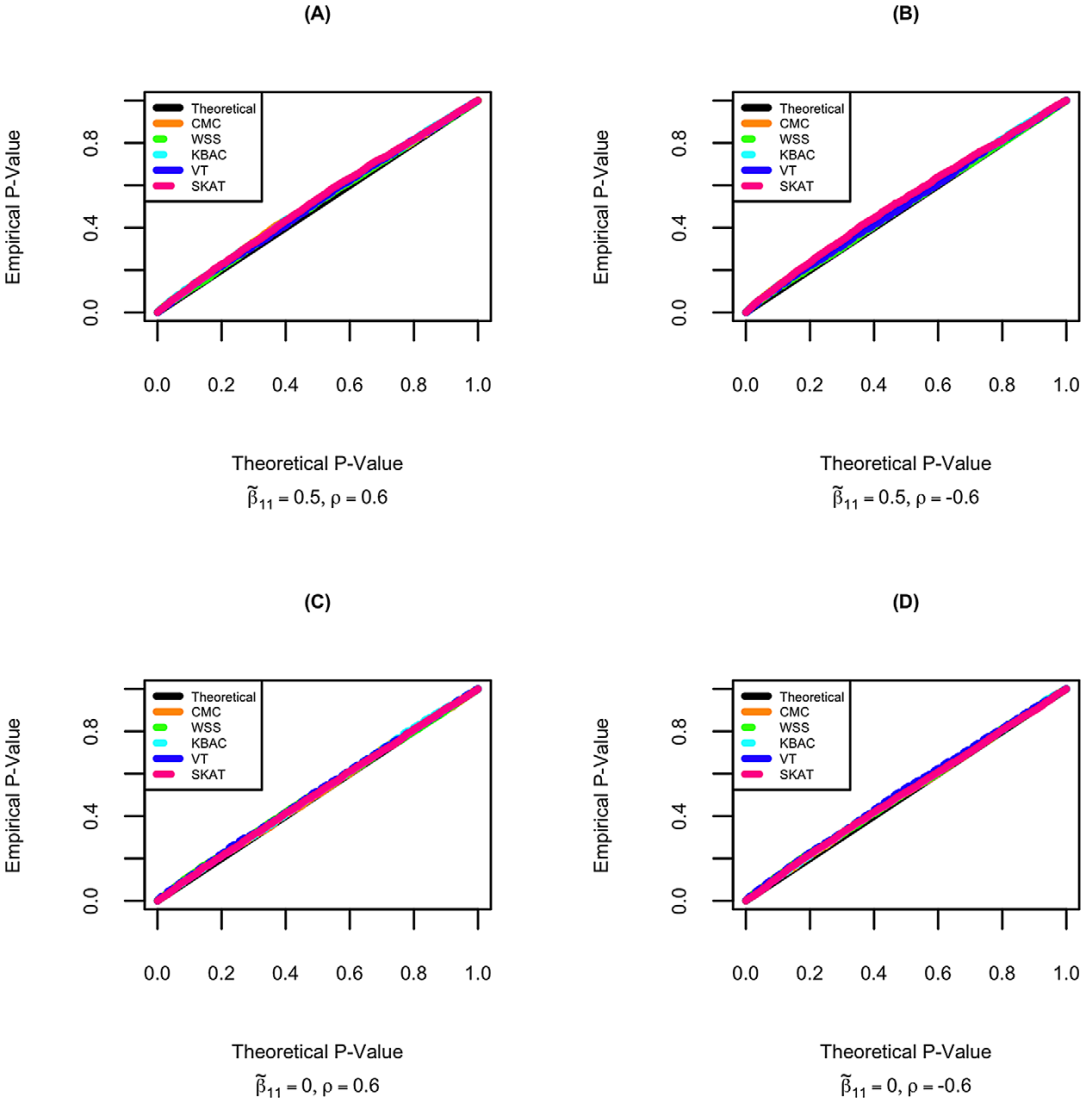
Quantile-Quantile plot of p-values for rare variant tests in STAR under the null hypothesis of no gene/secondary trait associations. Five tests were evaluated, i.e. CMC, WSS, KBAC, VT and SKAT. Empirical p-values for each test were plotted against their theoretical expectations. A variety of scenarios with different primary trait effects and trait residual correlations were examined, which include (A) 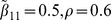; (B) 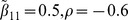; (C) 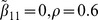 and (D) 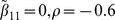. P-values were obtained with 5,000 permutations. Type I error was evaluated using 10,000 replicates. For each replicate, 5,000 individuals with extreme quantitative traits were selected from a cohort of 100,000.

Type I error for STAR was investigated when 1.) the gene region is neither associated with the primary trait nor the secondary trait. 2.) the gene region is associated only with the primary trait but not the secondary trait. The quantile-quantile plots of empirical p-values and their theoretical expectations are displayed for different rare variant tests. It can be seen that all tests incorporated in the STAR method have well controlled type I error. The p-values for the five tests are slightly conservative even when permutation is used to evaluate significance. This can occur when either the aggregate variant frequencies are low or the sample size is not sufficiently large. For example, when the primary trait effect is 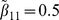 and residual correlation is 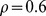, the type I errors for CMC, WSS, KBAC, VT and SKAT are respectively 0.048, 0.046, 0.042, 0.045 and 0.047 (Figure 1).

As a comparison, we also evaluated type I errors of linear regression analysis that ignores sample ascertainment mechanisms. When the gene region is not associated with the primary trait, type I errors for all rare variant tests are well controlled. However, if the gene region is also associated with the primary trait, the distribution of p-values under the null hypothesis is highly skewed and the type I errors for all tests are seriously inflated (Figure S3), which is concordant with our theoretical expectations.

### Evaluation of Power

**Figure 2:**
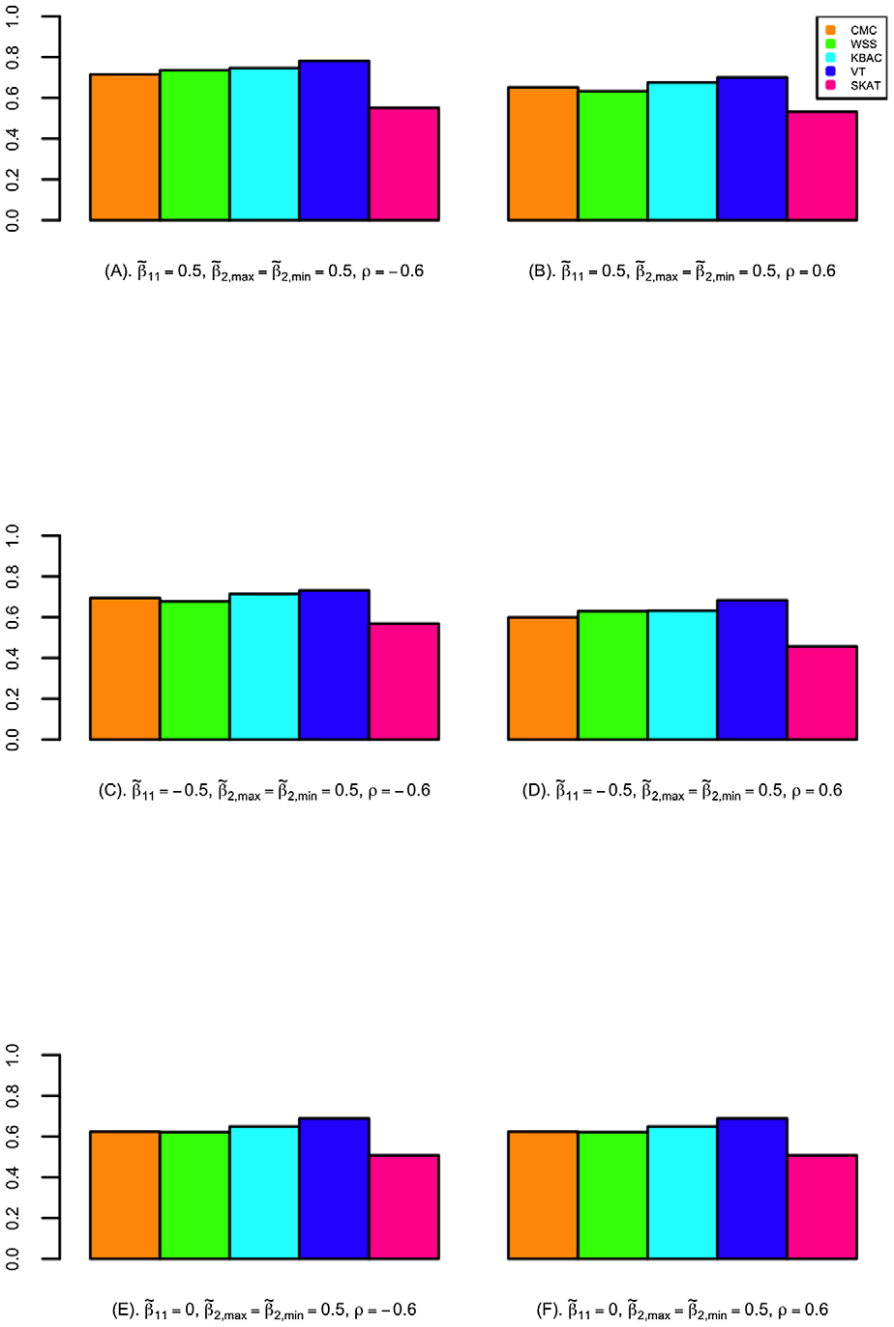
The power for detecting associations with secondary traits in selected samples. Power is calculated for CMC, WSS, KBAC, VT, and SKAT implemented in STAR framework. Secondary trait effects are assumed to be fixed and unidirectional with 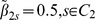. A variety of scenarios with different primary trait effects and trait residual correlations were examined, which include (A) 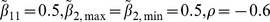; (B) 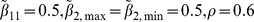; (C) 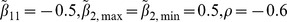; (D) 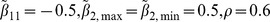; (E) 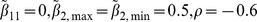 and (F) 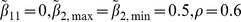. P-values were obtained with 5,000 permutations. Power was evaluated using 10,000 replicates for a significance level of 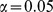. For each replicate, 5,000 individuals with extreme quantitative traits were selected from a cohort of 100,000 individuals.

**Figure 3:**
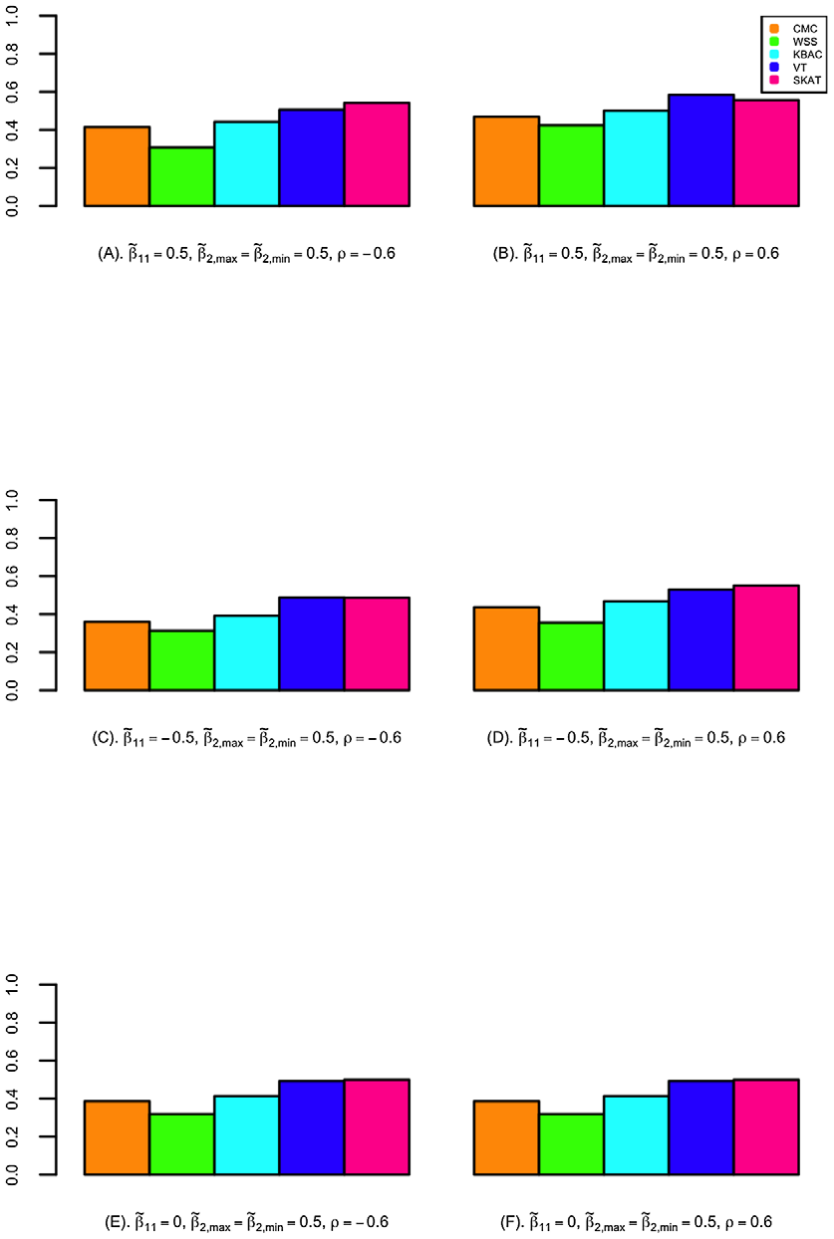
The power for detecting association with secondary traits in selected samples. Power is calculated for CMC, WSS, KBAC, VT, and SKAT implemented in STAR framework. Secondary trait effects are assumed to be bidirectional with fixed magnitude (i.e.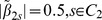), where 80% of the causal variants increase the mean secondary trait value and the other 20% decrease the mean secondary trait value. A variety of scenarios with different primary trait effects and trait residual correlations were examined, which include (A) 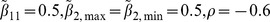; (B) 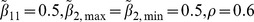; (C) 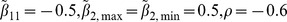; (D) 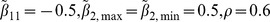; (E) 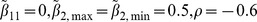 and (F) 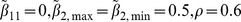. P-values were obtained with 5,000 permutations. Power was evaluated using 10,000 replicates for a significance level of 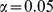. For each replicate, 5,000 individuals with extreme quantitative traits were selected from a cohort of 100,000 individuals.

The power of detecting secondary trait associations was compared for a variety of rare variant tests (Figure 2; Figure 3; and Figures S4, S5, S6, S7). Compared to the CMC method, the extended SKAT, WSS, KBAC and VT methods in STAR can be more powerful under a broad variety of models. For example, when the primary trait is associated with the gene region with effect 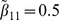 and trait residual correlation is 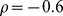, if causal variants have fixed unidirectional secondary trait effect, i.e. 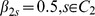, the power for WSS, KBAC and VT tests are respectively 73.5%, 74.1% and 78.1%, which all have greater power than the CMC (71.5%) (Figure 2). If the secondary trait effects are bidirectional, the power for the VT (50.6%) and SKAT (54.3%) tests are much higher than that of the CMC (41.4%), and the power for KBAC (44.1%) is also slightly greater than the power for the CMC (Figure 3).

VT can be more powerful than methods that use fixed variant frequency threshold, when the secondary trait effects are unidirectional. This is because using a fixed variant frequency threshold may result in the inclusion of higher frequency non-causal variants or the exclusion of more frequent causal variants from the analyses. For example, when the primary trait effect is 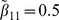 and trait residual correlation is 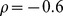, if the secondary trait effects are unidirectional and fixed with 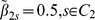, the power for VT is 78.1%, which is considerably higher than the power for the CMC (71.5%) (Figure 2). However, the difference in power between the best performing methods is small. For instance, when the primary trait effect is 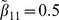 and trait residual correlation is 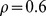, if the secondary trait effects are unidirectional and variable with 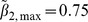 and 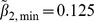, the power for VT is 85.9%, which is only 0.3% and 2.6% higher than the power for the WSS and KBAC.

The variance component score test SKAT is less powerful than burden tests when causal variant effects are unidirectional. For example, when 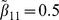, 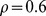, and the causal variant effects are unidirectional with 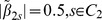, the power for SKAT is 53.1%, which is 24.3% lower than VT and 21.6% lower than KBAC (Figure 2). However, when the causal variant secondary trait effects are bidirectional, SKAT is among the most powerful methods. For instance, if the magnitudes of the causal variant effects are inversely correlated with MAFs, when 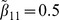, and 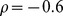, the power for SKAT is 63.2%, which is much greater than the power for CMC (49.2%), WSS (51.0%), and KBAC (54.4%) and slightly higher than the power for VT (60.3%) (Figure S5).

When the gene region is associated with both the primary and secondary traits, the power to detect secondary trait associations can be greater than when the gene region is only associated with the secondary trait. This is because variants with pleiotropic effects can be more enriched through extreme sampling. For example, when secondary trait effects are 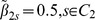, and residual correlation is 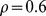, if the gene region is not associated with the primary trait, the power for CMC, WSS, KBAC, VT and SKAT are respectively 61.9%, 61.4%, 64.7%, 67.7% and 49.7% (Figure 2). However, if the gene region is also associated with primary trait with effect 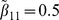, the power for the five tests increases to 65.3%, 63.3%, 67.7%, 70.1% and 53.1% respectively (Figure 2). Therefore, the power for detecting secondary trait associations can also be increased through sequencing samples with extreme primary trait values.

### Application of STAR to Meta-Analysis

The power and type I errors for STAR were evaluated for a simulated meta-analysis of three studies. As shown in (Figure S8), the empirical p-values and their theoretical expectations are well aligned on the quantile-quantile plot. Under a significance level of α = 0.05, the type I errors for the five rare variant tests are CMC (0.051), WSS (0.049), KBAC (0.049), VT (0.047), SKAT (0.051), which are well controlled. Due to the small sample size that is used, the type I errors for analyzing each individual study can still be slightly conservative. For example, in study 1, where causal variant effect for the primary trait is −0.5 and the correlation between the primary and secondary traits is 0.6, the type I errors for the five tests are respectively: CMC (0.046) WSS (0.044), KBAC (0.046), VT (0.047) and SKAT( 0.045).

We also evaluated the power of the STAR method under the alternative hypothesis (Figure S9). It can be seen that the power for meta-analysis is always higher than the power for each individual study, which highlights the benefit of combining multiple studies to detect associations with commonly measured traits.

### Analyses of SardiNIA Sequence Dataset

Sequence data from the SardiNIA project were analyzed to detect associations with multiple lipids and metabolic traits. First, association analyses were carried-out for the primary trait LDL levels (Table 1). In the original article by Sanna et al [1], extreme LDL values were dichotomized and association analyses were performed by comparing variant carrier frequencies between individuals sampled from opposite ends of the trait distribution. Only *APOB* was found to be nominally significantly associated with LDL (p-value 0.03). When QT values are analyzed instead of the dichotomized trait and more powerful association methods are used, the power to detect associations with the primary trait can be increased. For the association with *APOB*, the p-values for the five tests are 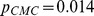, 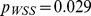, 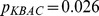, 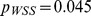, 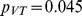, 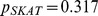. Additionally a significant association with *LDLR* that was not previously detected was also observed (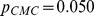, 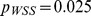, 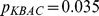, 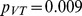, 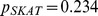). Among the tests that were used to analyze the association between *LDLR* and LDL, VT gives the smallest p-value. On the other hand, for the association with *APOB*, the score statistics from VT are maximized at the same MAF threshold as used by the other tests (i.e. 1%). In this case, the CMC test gives the most significant p-value.

**Table 1:** Association Analysis of *APOB*, *B3GA4*, *LDLR*, and *PCSK9* genes with LDL levels.

	CMC^a^^,^ c	WSS^a^^,^ c	KBAC^a^^,^ c	VT^b^^,^ c	SKAT^a^^,^ c
***APOB***	0.014*	0.029*	0.026*	0.045*	0.317
***B3GA4***	0.820	0.946	0.942	0.964	0.971
***LDLR***	0.050*	0.025*	0.035*	0.009^#^	0.234
***PCSK9***	0.272	0.299	0.381	0.491	0.491

a For CMC, WSS, KBAC, and SKAT, only variants with MAF≤1% were analyzed.

b For VT, variants with MAF≤5% were analyzed.

c The statistical significance of all tests was obtained empirically via 5,000 permutations. Nominally significant p-values are labeled with an asterisk. P-values that are significant after Bonferroni corrections are labeled with a pound sign.

Next we analyzed secondary trait associations with the four genes, i.e. *APOB*, *B3GA4*, *LDLR* and *PCSK9* (Table 2). One significant association, i.e. the association between *LDLR* and SysBP, is identified by CMC, WSS and KBAC after applying a Bonferroni correction for testing multiple genes and traits. The p-values for VT and SKAT are also nominally significant (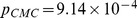, 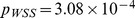, 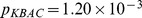, 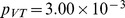, 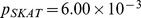). The score statistics in VT are maximized at the MAF cutoff 1%. In this scenario, the p-value of VT is not as significant as that of CMC, WSS and KBAC, because the burden test statistics are not increased at alternative frequency thresholds and a penalty for multiple testing is paid.

**Table 2:** Association Analyses of *APOB*, *B3GA4*, *LDLR*, and *PCSK9* genes.

Gene	Trait	CMC^a^^,^ c	WSS^a^^,^ c	KBAC^a^^,^ c	VT^b^^,^ c	SKAT^a^^,^ c
*APOB*	TCL	3.07E-01	6.04E-01	6.75E-01	2.76E-01	8.88E-01
*APOB*	HDL	7.00E-01	9.71E-01	5.35E-01	9.21E-01	6.30E-01
*APOB*	BMI	5.98E-01	2.57E-01	7.05E-01	5.22E-01	2.29E-01
*APOB*	DiasBP	9.10E-01	1.71E-01	1.52E-01	2.22E-01	3.79E-01
*APOB*	SysBP	7.54E-01	6.74E-01	5.37E-01	9.22E-01	7.69E-01
*APOB*	TG	8.76E-01	3.60E-01	2.46E-01	4.16E-01	7.06E-01
*APOB*	INSULIN	8.30E-01	4.85E-01	4.07E-01	5.96E-01	1.68E-01
*B3GA4*	TCL	6.67E-01	8.18E-01	6.97E-01	3.93E-01	1.54E-01
*B3GA4*	HDL	8.71E-01	2.78E-01	2.21E-01	4.14E-01	6.90E-02
*B3GA4*	BMI	3.81E-01	7.72E-01	7.66E-01	9.50E-01	9.84E-01
*B3GA4*	DiasBP	5.63E-01	8.10E-01	8.58E-01	4.41E-01	5.29E-01
*B3GA4*	SysBP	5.39E-01	9.47E-01	9.22E-01	8.26E-01	8.62E-01
*B3GA4*	TG	5.60E-01	9.22E-01	9.14E-01	6.12E-01	4.98E-01
*B3GA4*	INSULIN	5.14E-01	9.74E-01	9.79E-01	9.73E-01	9.85E-01
*LDLR*	TCL	2.31E-02*	3.60E-02*	2.90E-02*	4.90E-02*	8.93E-01
*LDLR*	HDL	1.13E-01	1.59E-01	2.35E-01	4.19E-01	4.61E-01
*LDLR*	BMI	1.01E-01	2.62E-01	1.94E-01	3.74E-01	7.41E-01
*LDLR*	DiasBP	1.64E-02*	2.70E-02*	2.50E-02*	4.70E-02*	2.33E-01
*LDLR*	SysBP	9.14E-04^#^	3.08E-04^#^	1.20E-03^#^	3.00E-03^#^	6.00E-03^#^
*LDLR*	TG	4.73E-01	9.21E-01	9.64E-01	9.88E-01	9.97E-01
*LDLR*	INSULIN	3.76E-01	7.91E-01	7.77E-01	4.88E-01	9.67E-01
*PCSK9*	TCL	1.98E-02*	4.80E-02^*^	2.30E-02*	1.52E-01	9.22E-01
*PCSK9*	HDL	4.98E-02*	6.70E-02	5.80E-02	1.44E-01	2.33E-01
*PCSK9*	BMI	3.85E-01	6.81E-01	7.27E-01	4.11E-01	8.73E-01
*PCSK9*	DiasBP	4.24E-01	8.03E-01	8.42E-01	1.18E-01	5.05E-01
*PCSK9*	SysBP	2.76E-01	5.67E-01	5.79E-01	1.28E-01	7.43E-01
*PCSK9*	TG	3.29E-01	5.25E-01	6.53E-01	6.56E-01	6.31E-01
*PCSK9*	INSULIN	7.53E-01	6.15E-01	4.83E-01	1.12E-01	5.46E-01

Secondary traits, total cholesterol levels (TCL), high density lipoprotein (HDL), body mass index (BMI), diastolic blood pressure (DiasBP), systolic blood pressure (SysBP), triglyceride (TG) and insulin levels (INSULIN) were studied.

a For CMC, WSS, KBAC, and SKAT, variants with MAF≤1% were analyzed.

b For VT, variants with MAF≤5% were analyzed.

c Statistical significance for all tests was obtained empirically via 5,000 permutations. Nominally significant p-values are labeled with an asterisk, while the associations that are significant after Bonferroni corrections are labeled with a pound sign.

It is interesting to note that *LDLR* is associated with both the primary trait LDL and the secondary trait SysBP, which are correlated with a coefficient of 0.145 (Table S1). It is possible that a portion of the rare variants in *LDLR* have pleiotropic effects and are enriched in the dataset via selective sampling on the primary trait, which increase the power for detecting secondary trait associations.

We also compared the analysis using STAR and standard linear regressions (Table S2). Due to the small sample sizes that are used, we did not observe an excess of false positive signals for the naïve linear regression analysis. However, we noted that for the association between *LDLR* and SysBP, the p-values from STAR are smaller. In addition, for the associations between *LDLR*, *PCSK9* and TCL that were previously implicated in genome-wide association studies [30], the p-values from STAR are also more significant.

## Discussion

In this article, we present a likelihood model which can be used to analyze secondary trait associations in selected samples. The method corrects for the bias in the distribution of the secondary traits induced by selective sampling. All rare variant association analysis methods can be extended within the STAR framework. STAR makes it possible to apply more powerful rare variant association tests for the analysis of secondary trait and allows jointly analyzing cohorts that were ascertained for different primary traits. The power for detecting associations with secondary traits can be greatly enhanced. In addition to performing gene-based association analysis, the STAR method and STARSEQ software can also be applied to detect single variant associations (data not shown).

Currently, many sequence based genetic studies are being performed to detect associations with complex traits. Due to the high cost of sequencing, the sample sizes for many of these studies are small. It was previously shown that in order to have sufficient power (e.g. >80%) to detect association with rare variants in an exome-wide study, in some cases it is necessary to sequence at least 10,000 samples with extreme traits from a cohort of 100,000 [25]. However, both the cohort size and the cost of sequencing exceed the capacity of most studies. Therefore to increase power it is important that multiple studies can be jointly analyzed. The STAR method is particularly useful, since cohorts that are ascertained for diverse primary traits using different study designs can be jointly analyzed.

Previously CMC and GRANVIL tests were extended for analyzing secondary traits with p-value being evaluated analytically [21]. Many of the rare variant association methods implemented in STAR can be more powerful than CMC and GRANVIL. In fact, despite being computationally efficient, CMC and GRANVIL can be underpowered when a large portion of the variants in the gene region are non-causal or when the genetic effects of causal variants are bidirectional. Other methods such as SKAT may perform better in these scenarios. In addition, through assigning weights to different variant sites, variants that are potentially causal can be assigned higher weights, which can help to distinguish causal from non-causal variants. When variable selection based methods are used, the set of variants where the Z-score statistics are maximized can be selected and tested. These methods can be more robust against the inclusion of non-causal variant in the analysis, and also potentially be more powerful than CMC and GRANVIL methods even after adjusting for multiple comparisons.

Permutation algorithm is often a necessary ingredient for rare variant association tests. Even if asymptotic approximations exist for some rare variant association tests such as SKAT and CMC, they may not be accurate and type I errors may be inflated or deflated [23]. This is because the asymptotic distribution for the test statistic can be affected by the number of rare variant sites and variant site frequency spectrums [17], [23]. In practice, there can be considerable variation in the number of variant sites and frequencies within a gene region [31]. It is possible that an asymptotically valid test has inflated or deflated type I errors when genetic regions with only a few variant alleles are analyzed. Therefore, when a significant analytical p-value is obtained, it is necessary to empirically confirm the result using permutation.

Under the STAR framework, we compared the power of several rare variant tests for analyzing secondary traits in selected samples. It is clear from our comparisons that when causal variants have unidirectional effects, burden tests perform better than SKAT. However, when variants with effects in opposite directions are present, SKAT can be more powerful than burden test based methods. Given that the goal of the article is to introduce a method for analyzing secondary traits in selected samples, rather than to compare different rare variant tests, our simulations are not as comprehensive as some existing reviews, such as Basu and Pan [32] and Ladouceur et al [33]. However, based upon the simulation scenarios that we considered, it is clear that the power for detecting associations can be greatly improved through the STAR model. In addition, our conclusions for comparing multiple rare variant tests are also compatible with the comprehensive reviews, in that there is not a consistently most powerful rare variant test and the difference in power between top performing methods is usually small. In addition to the simulation experiments, it is also important to examine and compare the performance of different methods in large scale sequencing studies, such as the NHLBI-ESP.

In the analysis of the SardiNIA dataset, we adjusted the blood pressure for individuals undergoing antihypertensive therapy. The rank of sample blood pressure traits was only slightly changed after the adjustment. Given that we quantile-normalized the trait prior to the association analysis, the impact of the adjustment on the result is very minimal. In order to evaluate the robustness of the results, we also analyzed the associations with blood pressure when no adjustments were made, and the results are very similar (data not shown). A significant association was identified between rare variants in *LDLR* and secondary trait SysBP, where carriers of rare variants in the *LDLR* gene tend to have lower SysBP levels. In fact, the *LDLR* gene has also been shown to be strongly associated with reductions of SysBP among the patients that receive atenolol, an antihypertensive drug [26]. These discoveries imply that variants in the *LDLR* gene may influence the etiology of SysBP. *LDLR* is potentially an important gene target for blood pressure treatment. In order to replicate signals [22] that were found in the SardiNIA cohort, many current large scale sequencing studies can be considered, such as the NHLBI-ESP etc. In addition to replicating associations, there is also great scientific interest in exploring whether rare causal variants identified in a founder population are identical to those from out-bred populations [34].

With the large scale application of next generation sequencing to study complex traits, samples from many existing cohorts will be sequenced. There can be insufficient power for analyzing associations in each individual study. It would be highly beneficial if samples from multiple cohorts can be combined for analyzing commonly measured traits. STAR is thus important and will greatly accelerate the process of identifying genes involved in complex trait etiology.

## Supporting Information

Figure S1Conditional distribution of the secondary trait for individuals with extreme primary trait values in the upper and lower 5% tails. The density function of the secondary traits is plotted when the primary trait effect 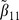 is 0.5 and the trait residual correlation 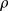 is 0.6.(TIF)

Figure S2Biases of the secondary trait effects due to extreme sampling under the null hypothesis. The bias in the secondary trait effects i.e. 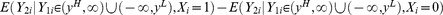 is plotted against the primary trait effects and trait residual correlations. In panel (A), the bias is plotted when the primary trait effect 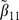 is 0.5, and the trait residual correlation 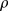 varies between −0.8 and 0.8. In panel (B), the bias is plotted when the trait residual correlation 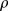 is 0.6, and the primary trait effect 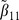 varies between −3 and 3.(TIF)

Figure S3Quantile-Quantile plot of p-values for rare variant tests in linear regression models under the null hypothesis of no gene/secondary trait associations. Sample ascertainment mechanism was ignored in the linear regression analysis. Five tests were evaluated, i.e. CMC, WSS, KBAC, VT and SKAT. Empirical p-values for each test were plotted against their theoretical expectations. A variety of scenarios with different primary trait effects and trait residual correlations were examined, which include (A) 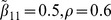; (B) 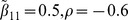; (C) 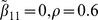 and (D) 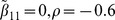. P-values were obtained with 5,000 permutations. Type I error was evaluated using 10,000 replicates. For each replicate, 5,000 individuals with extreme quantitative traits were selected from a cohort of 100,000.(TIF)

Figure S4The power for detecting association with secondary traits in selected samples. The power is shown for CMC, WSS, KBAC, VT, and SKAT implemented in the STAR framework. It is assumed that the secondary trait effects for causal variants are unidirectional, and their magnitudes are inversely proportional to the minor allele frequencies with 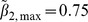 and 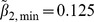. A variety of scenarios with different primary trait effects and trait residual correlations were examined, which include (A) 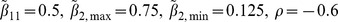; (B) 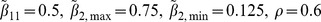; (C) 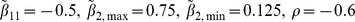; (D) 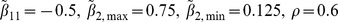; (E) 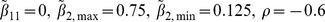 and (F) 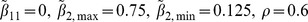. P-values were obtained empirically via 5,000 permutations. Power was evaluated using 10,000 replications for a significance level of 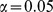. For each replicate, 5,000 individuals with extreme quantitative traits were selected from a cohort of 100,000 individuals.(TIF)

Figure S5The power for detecting association with secondary traits in selected samples. The power is shown for CMC, WSS, KBAC, VT, and SKAT implemented in STAR framework. It is assumed that 80% of the causal variants increase the mean secondary trait value, and the remaining variants decrease the mean secondary trait value. The magnitudes of the secondary trait effects are inversely proportional to the minor allele frequencies, with 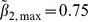 and 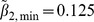. A variety of scenarios with different primary trait effects and trait residual correlations were examined, which include (A) 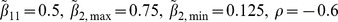; (B) 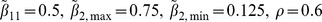; (C) 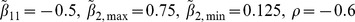; (D) 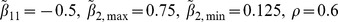; (E) 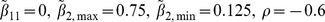 and (F) 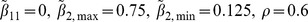. P-values were obtained empirically via 5,000 permutations. Power was evaluated using 10,000 replicates for a significance level of 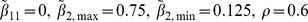. For each replicate, 5,000 individuals with extreme quantitative traits were selected from a cohort of 100,000 individuals.(TIF)

Figure S6The power for detecting associations with secondary traits in selected samples. Power is shown for CMC, WSS, KBAC, VT, and SKAT implemented in STAR framework. Secondary trait effects are assumed to be fixed and unidirectional with 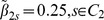. A variety of scenarios with different primary trait effects and trait residual correlations were examined, which include (A) 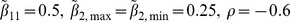; (B) 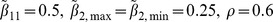; (C) 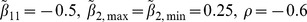; (D) 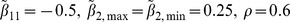; (E) 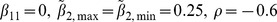 and (F) 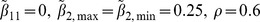. P-values were obtained empirically via 5,000 permutations. Power was evaluated using 10,000 replicates for a significance level of 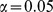. For each replicate, 5,000 individuals with extreme quantitative traits were selected from a cohort of 100,000 individuals.(TIF)

Figure S7The power for detecting association with secondary traits in selected samples. Power is shown for CMC, WSS, KBAC, VT, and SKAT implemented in STAR framework. It is assumed that secondary trait effects are bidirectional with fixed magnitude (i.e.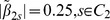), where 80% of the causal variants increase the mean secondary trait value and the other 20% decrease the mean secondary trait value. A variety of scenarios with different primary trait effects and trait residual correlations were examined, which include (A) 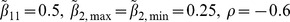; (B) 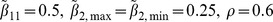; (C) 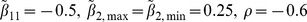; (D) 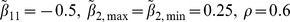; (E) 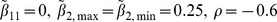 and (F) 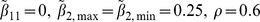. P-values were obtained empirically via 5,000 permutations. Power was evaluated using 10,000 replicates for a significance level of 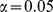. For each replicate, 5,000 individuals with extreme quantitative traits were selected from a cohort of 100,000 individuals.(TIF)

Figure S8 Quantile-Quantile plot for meta-analysis p-values under the null hypothesis. Meta-analysis for three studies was simulated. The primary trait in each study is assumed to be different and a common secondary trait is measured in all studies. The gene region is not associated with the secondary trait. In the first study, the gene region is associated with the primary trait, and causal variants have an effect of −0.5. The correlation between the primary and secondary traits is 0.6. In the second study, the primary trait is also associated with the gene region, and causal variants have an effect of 0.25. The primary and secondary traits are correlated with coefficient 0.4. In the third study, the gene region is not associated with the primary trait, and the correlation between the primary and secondary traits is −0.2. CMC, WSS, KBAC, VT and SKAT were used to detect associations. In each study, a pool of 50,000 samples was simulated and 2,500 individuals with extreme primary trait were selected and analyzed. P-values for all rare variant tests were obtained based upon 5,000 permutations. The empirical distribution of p-values was obtained using 10,000 replicates.
(TIF)

Figure S9 Power for meta-analysis using CMC, WSS, KBAC, VT and SKAT. Meta-analysis for three studies was simulated. The primary trait in each study is assumed to be different and a common secondary trait is measured in all studies. Power for the five tests was displayed when (A) causal variants have unidirectional effect of 0.5, and (B) causal variants have bidirectional effects, i.e. 80% of the causal variants have effect 0.5 and the other 20% have effect −0.5. In the first study, the gene region is associated with the primary trait, and causal variants have an effect of −0.5. The correlation between the primary and secondary traits is 0.6. In the second study, the primary trait is also associated with the gene region, and causal variants have an effect of 0.25. The primary and secondary traits are correlated with coefficient 0.4. In the third study, the gene region is not associated with the primary trait, and the correlation between the primary and secondary traits is −0.2. In each study, a different pool of 50,000 samples was simulated and 2,500 individuals with extreme primary trait were selected and analyzed. P-values for all rare variant tests were obtained based upon 5,000 permutations. The power for analyzing each individual study and meta-analysis was evaluated using 10,000 replicates.
(TIF)

Table S1 Correlations of phenotypes from the SardiNIA cohort. Eight traits that were analyzed for associations are included, i.e. high density lipoprotein (HDL), low density lipoprotein (LDL), triglyceride (TG), total cholesterol levels (TCL), diastolic blood pressure (DiasBP), systolic blood pressure (SysBP), insulin levels (INSULIN), and body mass index (BMI). Correlations were estimated using 2044 unrelated individuals extracted from the SandiNIA pedigrees.
(DOC)

Table S2 Analysis of secondary trait associations using standard linear regression. Sample ascertainment mechanisms were ignored in the analysis. Seven secondary traits were analyzed, including total cholesterol levels (TCL), high density lipoprotein (HDL), body mass index (BMI), diastolic blood pressure (DiasBP), systolic blood pressure (SysBP), triglyceride (TG) and insulin levels (INSULIN). Gene-based association analysis was performed using CMC, WSS, KBAC, VT and SKAT.
(DOC)

Text S1 Extension of Kernel Based Adaptive Cluster to the Analysis of Quantitative Traits.
(PDF)

Text S2 Biases of Naïve Inferences of Secondary Trait Associations in Selected Samples.
(PDF)

Text S3 Details for the Null Likelihood Model.
(PDF)

Text S4 Practical Issues for Inferences under the Ascertainment Corrected Likelihood.
(PDF)

Text S5 Constructing Variance Component Score Tests from Ascertainment Corrected Likelihood.
(PDF)
