# Supplementary figures and images for: A Unified Method for Detecting Secondary Trait Associations with Rare Variants: Application to Sequence Data
Source: PLoS Genet. 2012 Nov 15;8(11):e1003075. doi: 10.1371/journal.pgen.1003075 (PMC3499373; doi:10.1371/journal.pgen.1003075)

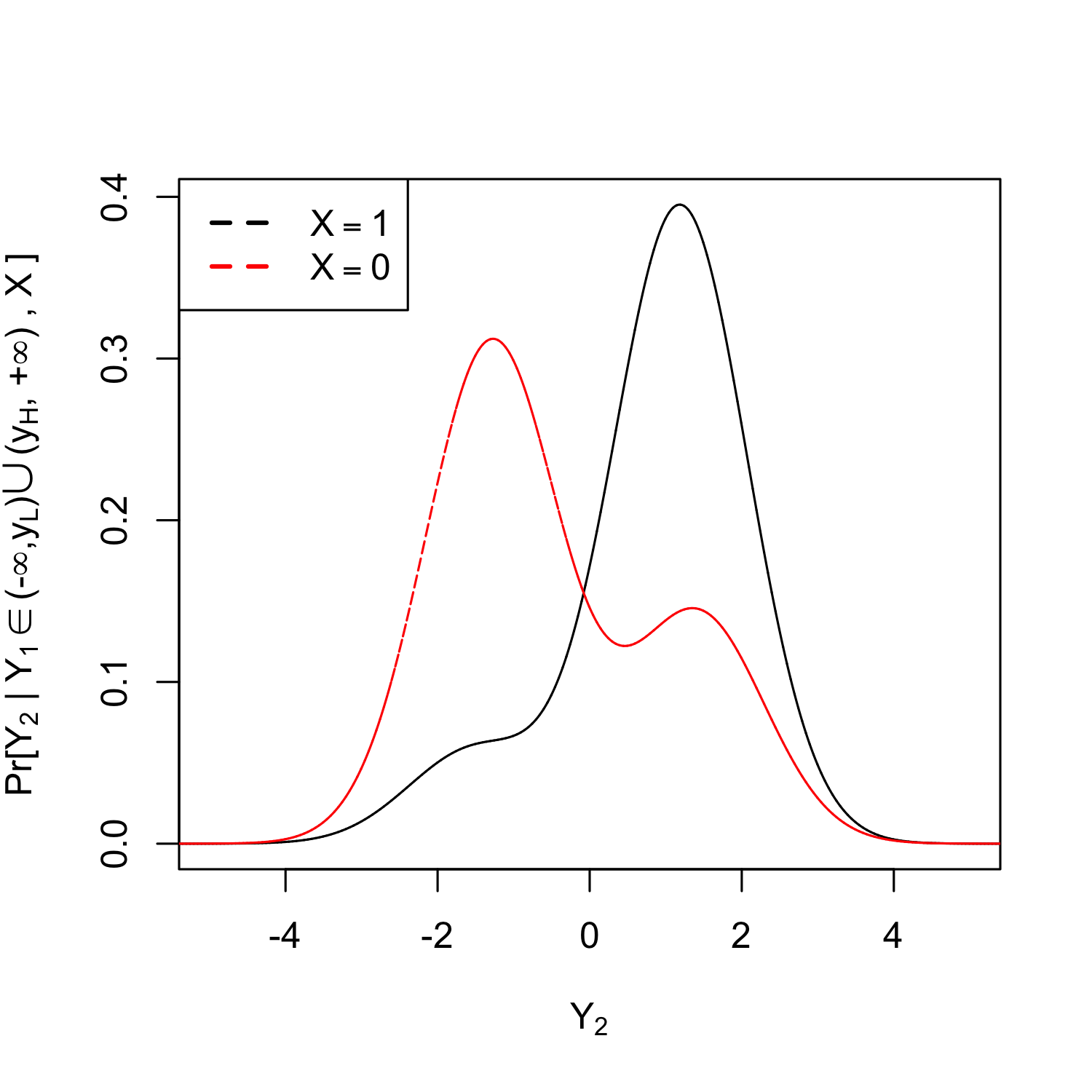

Supplement: Figure S1 — Conditional distribution of the secondary trait for individuals with extreme primary trait values in the upper and lower 5% tails. The density function of the secondary traits is plotted when the primary trait effect is 0.5 and the trait residual correlation is 0.6. (TIF) [file pgen.1003075.s001.tif]

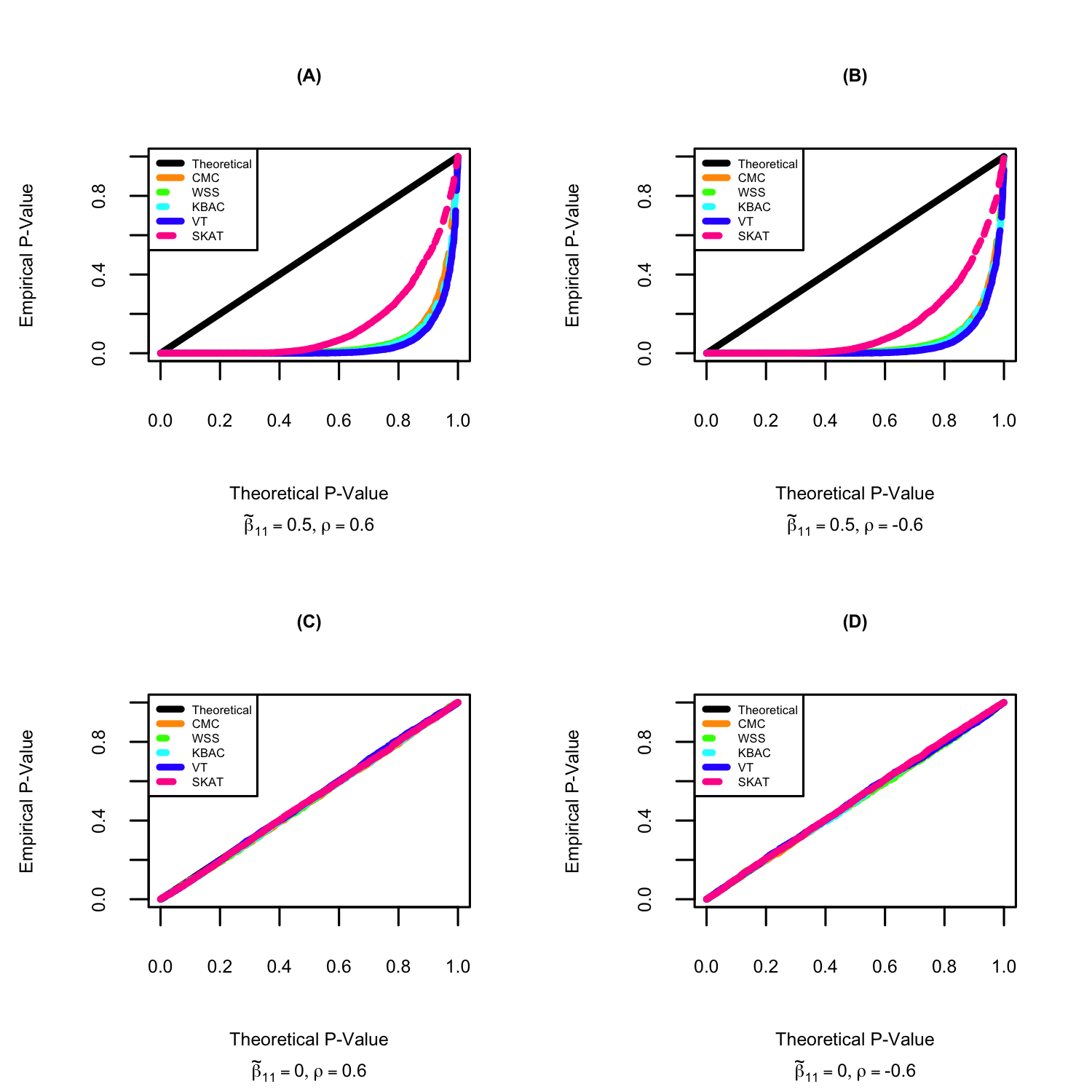

Supplement: Figure S3 — Quantile-Quantile plot of p-values for rare variant tests in linear regression models under the null hypothesis of no gene/secondary trait associations. Sample ascertainment mechanism was ignored in the linear regression analysis. Five tests were evaluated, i.e. CMC, WSS, KBAC, VT and SKAT. Empirical p-values for each test were plotted against their theoretical expectations. A variety of scenarios with different primary trait effects and trait residual correlations were examined, which include (A) ; (B) ; (C) and (D) . P-values were obtained with 5,000 permutations. Type I error was evaluated using 10,000 replicates. For each replicate, 5,000 individuals with extreme quantitative traits were selected from a cohort of 100,000. (TIF) [file pgen.1003075.s003.tif]

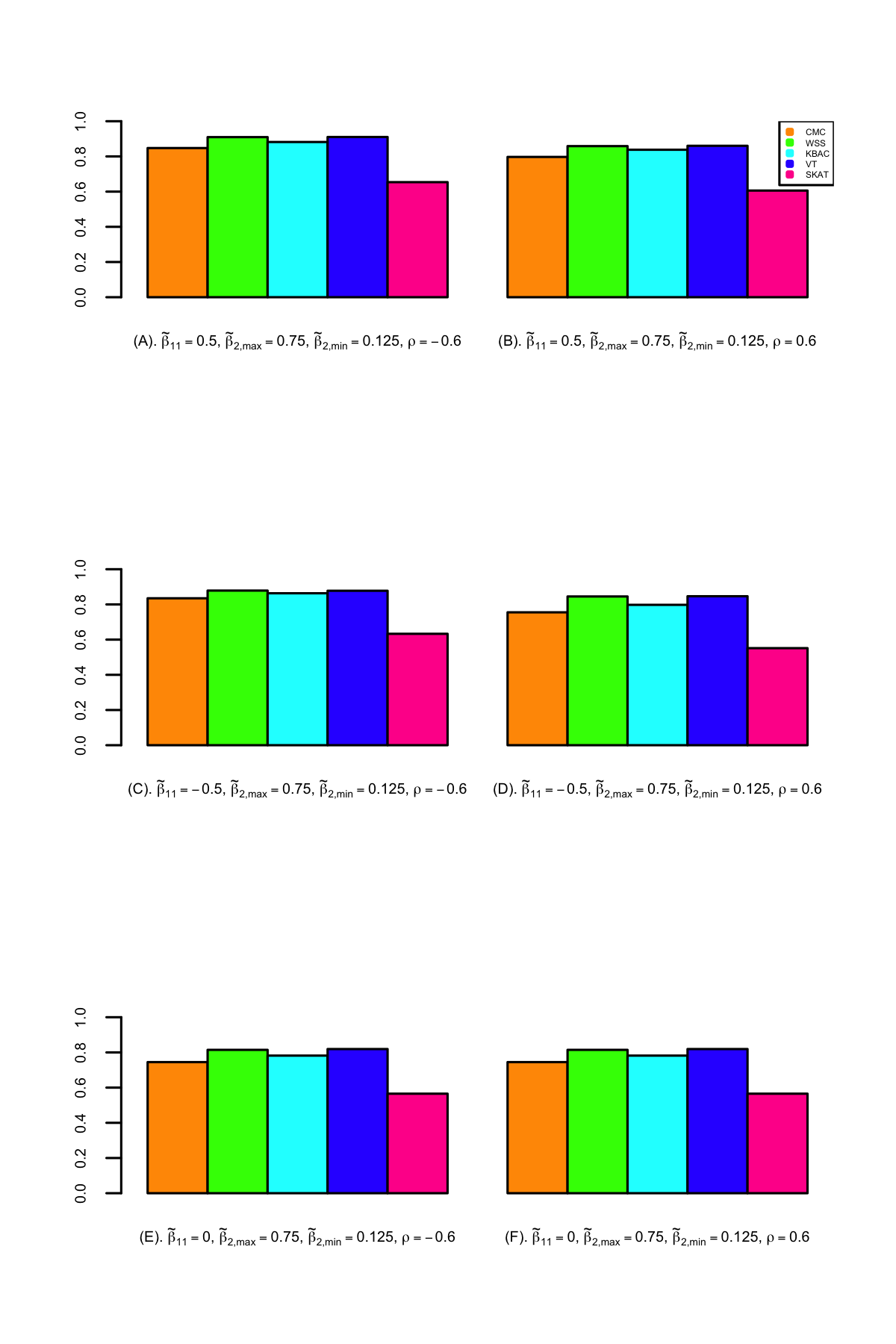

Supplement: Figure S4 — The power for detecting association with secondary traits in selected samples. The power is shown for CMC, WSS, KBAC, VT, and SKAT implemented in the STAR framework. It is assumed that the secondary trait effects for causal variants are unidirectional, and their magnitudes are inversely proportional to the minor allele frequencies with and . A variety of scenarios with different primary trait effects and trait residual correlations were examined, which include (A) ; (B) ; (C) ; (D) ; (E) and (F) . P-values were obtained empirically via 5,000 permutations. Power was evaluated using 10,000 replications for a significance level of . For each replicate, 5,000 individuals with extreme quantitative traits were selected from a cohort of 100,000 individuals. (TIF) [file pgen.1003075.s004.tif]

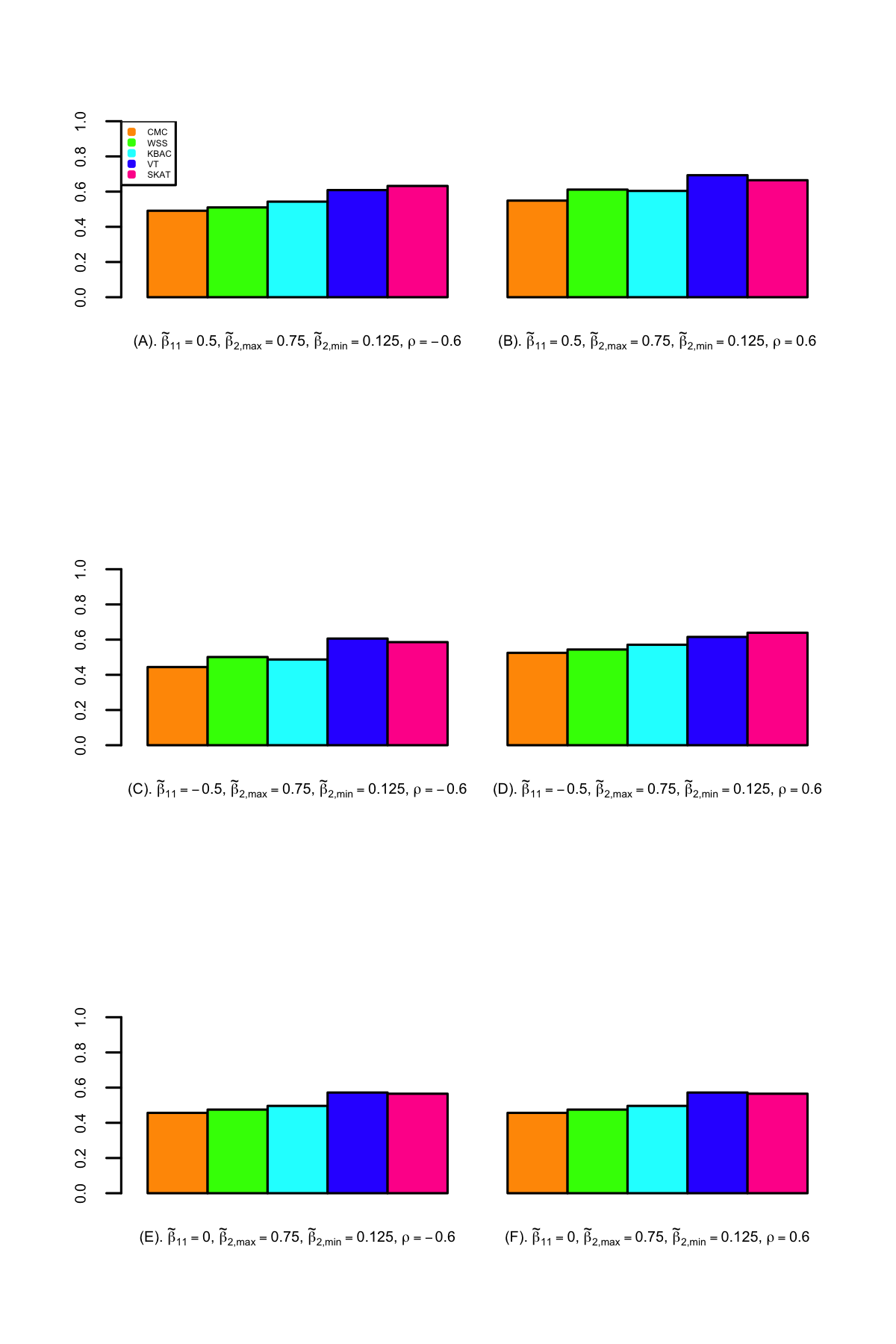

Supplement: Figure S5 — The power for detecting association with secondary traits in selected samples. The power is shown for CMC, WSS, KBAC, VT, and SKAT implemented in STAR framework. It is assumed that 80% of the causal variants increase the mean secondary trait value, and the remaining variants decrease the mean secondary trait value. The magnitudes of the secondary trait effects are inversely proportional to the minor allele frequencies, with and . A variety of scenarios with different primary trait effects and trait residual correlations were examined, which include (A) ; (B) ; (C) ; (D) ; (E) and (F) . P-values were obtained empirically via 5,000 permutations. Power was evaluated using 10,000 replicates for a significance level of . For each replicate, 5,000 individuals with extreme quantitative traits were selected from a cohort of 100,000 individuals. (TIF) [file pgen.1003075.s005.tif]

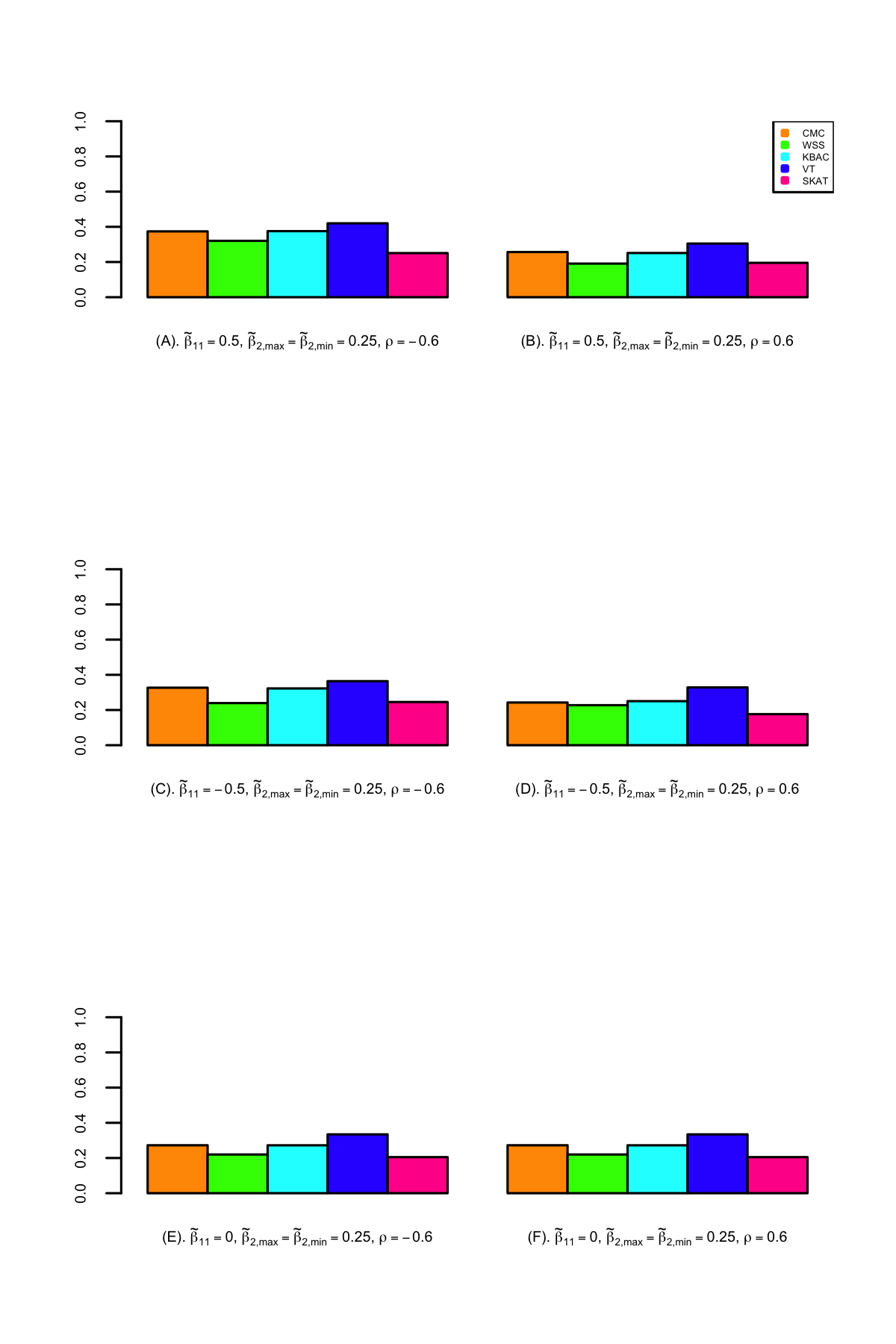

Supplement: Figure S6 — The power for detecting associations with secondary traits in selected samples. Power is shown for CMC, WSS, KBAC, VT, and SKAT implemented in STAR framework. Secondary trait effects are assumed to be fixed and unidirectional with . A variety of scenarios with different primary trait effects and trait residual correlations were examined, which include (A) ; (B) ; (C) ; (D) ; (E) and (F) . P-values were obtained empirically via 5,000 permutations. Power was evaluated using 10,000 replicates for a significance level of . For each replicate, 5,000 individuals with extreme quantitative traits were selected from a cohort of 100,000 individuals. (TIF) [file pgen.1003075.s006.tif]

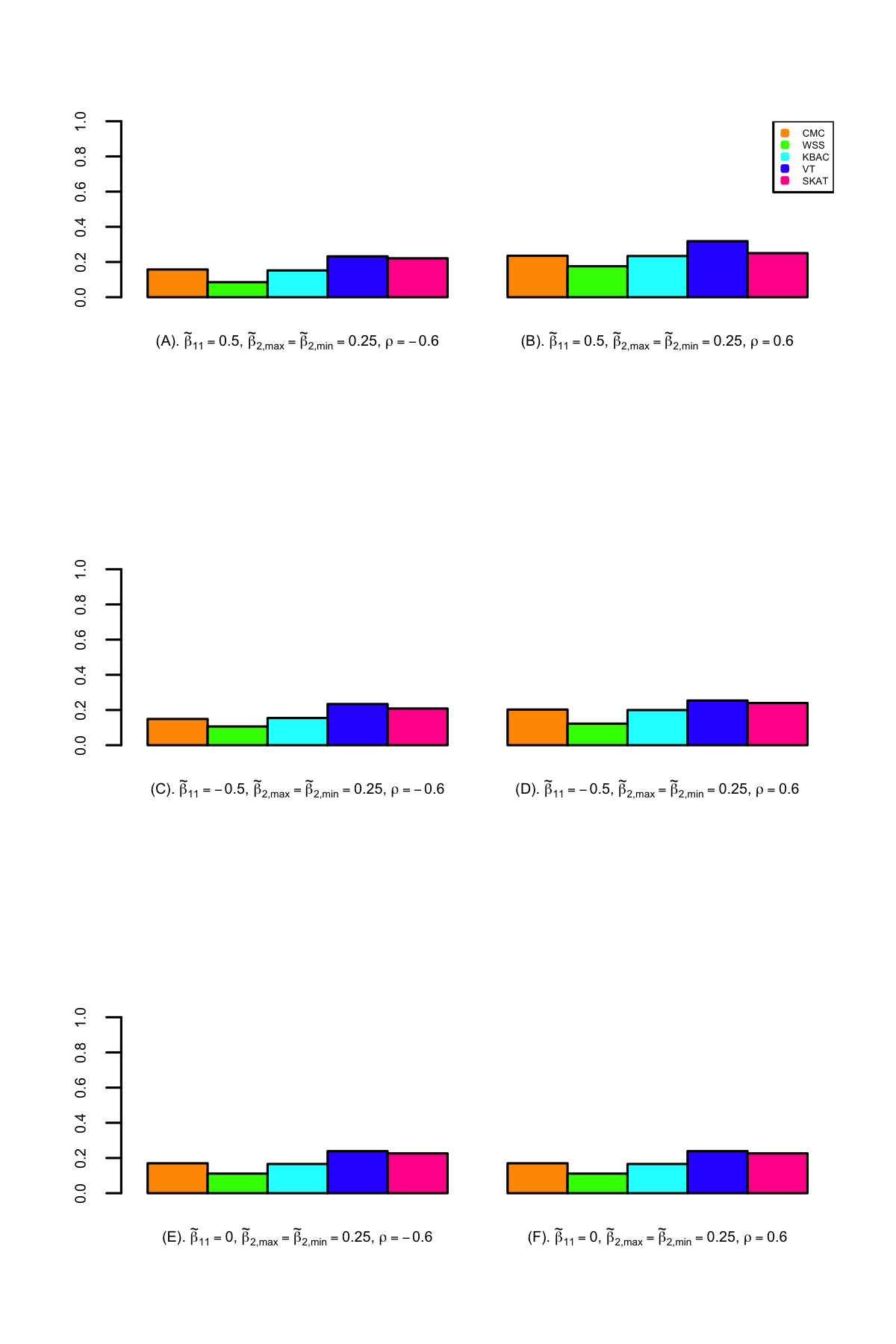

Supplement: Figure S7 — The power for detecting association with secondary traits in selected samples. Power is shown for CMC, WSS, KBAC, VT, and SKAT implemented in STAR framework. It is assumed that secondary trait effects are bidirectional with fixed magnitude (i.e.), where 80% of the causal variants increase the mean secondary trait value and the other 20% decrease the mean secondary trait value. A variety of scenarios with different primary trait effects and trait residual correlations were examined, which include (A) ; (B) ; (C) ; (D) ; (E) and (F) . P-values were obtained empirically via 5,000 permutations. Power was evaluated using 10,000 replicates for a significance level of . For each replicate, 5,000 individuals with extreme quantitative traits were selected from a cohort of 100,000 individuals. (TIF) [file pgen.1003075.s007.tif]

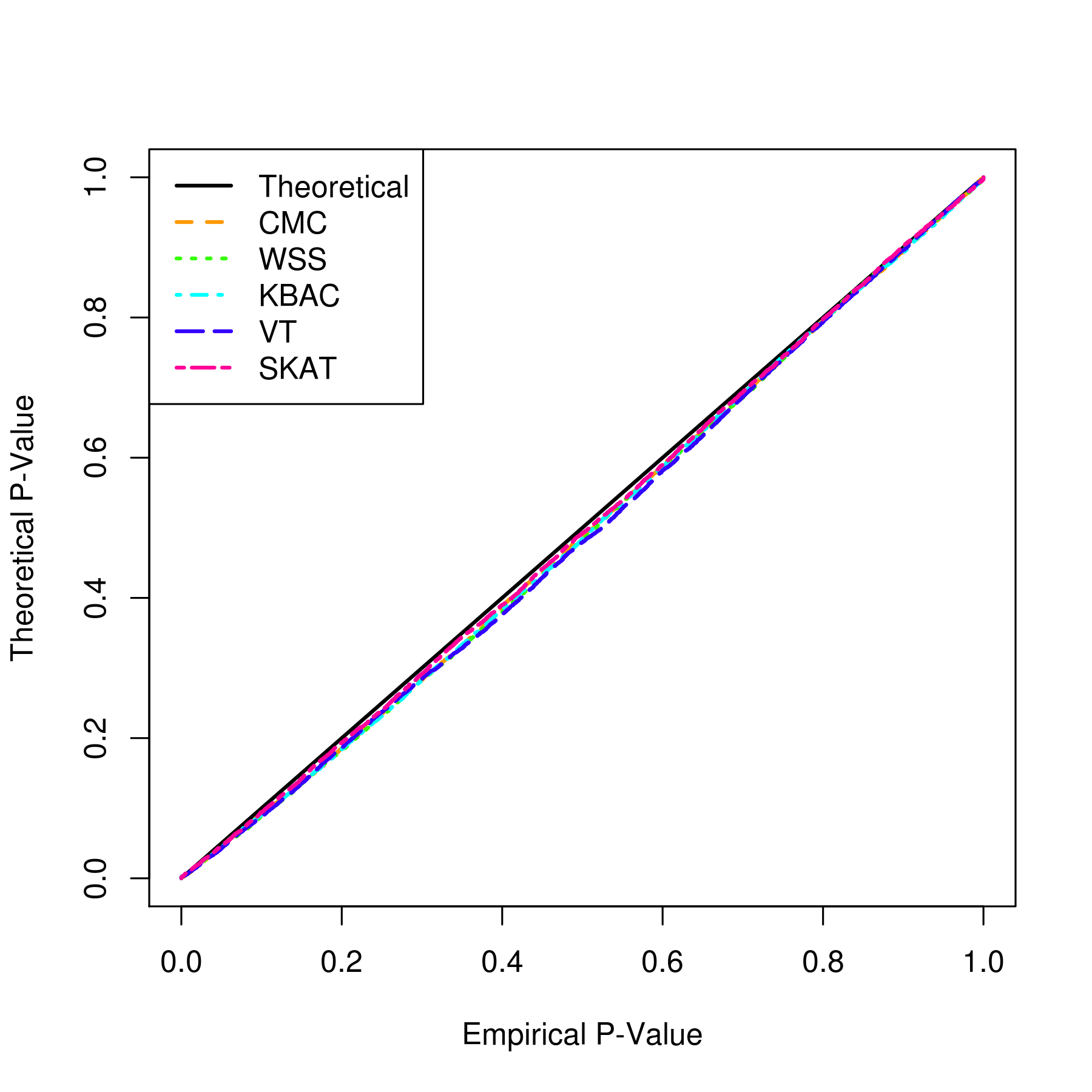

Supplement: Figure S8 — Quantile-Quantile plot for meta-analysis p-values under the null hypothesis. Meta-analysis for three studies was simulated. The primary trait in each study is assumed to be different and a common secondary trait is measured in all studies. The gene region is not associated with the secondary trait. In the first study, the gene region is associated with the primary trait, and causal variants have an effect of −0.5. The correlation between the primary and secondary traits is 0.6. In the second study, the primary trait is also associated with the gene region, and causal variants have an effect of 0.25. The primary and secondary traits are correlated with coefficient 0.4. In the third study, the gene region is not associated with the primary trait, and the correlation between the primary and secondary traits is −0.2. CMC, WSS, KBAC, VT and SKAT were used to detect associations. In each study, a pool of 50,000 samples was simulated and 2,500 individuals with extreme primary trait were selected and analyzed. P-values for all rare variant tests were obtained based upon 5,000 permutations. The empirical distribution of p-values was obtained using 10,000 replicates. (TIF) [file pgen.1003075.s008.tif]

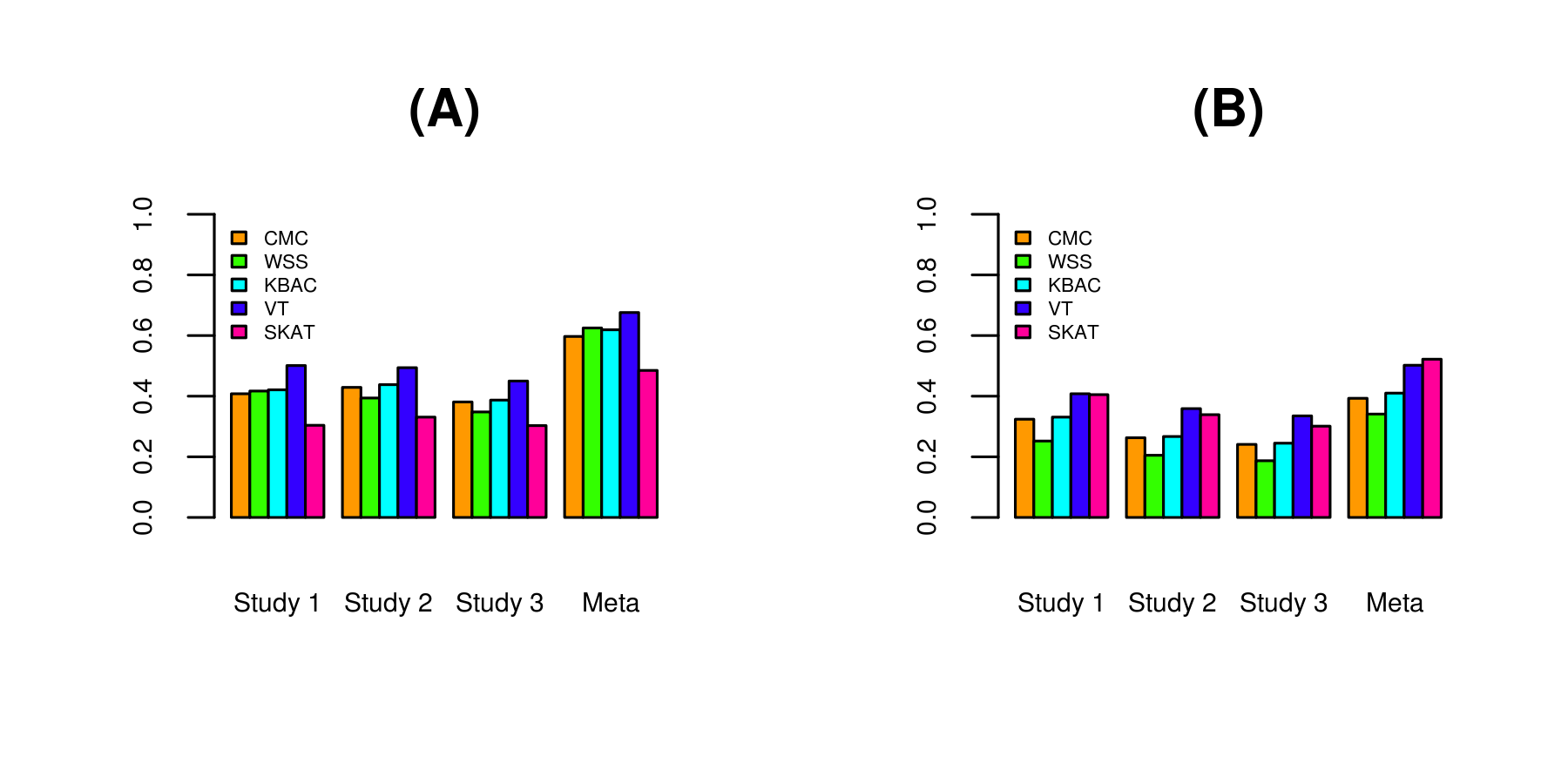

Supplement: Figure S9 — Power for meta-analysis using CMC, WSS, KBAC, VT and SKAT. Meta-analysis for three studies was simulated. The primary trait in each study is assumed to be different and a common secondary trait is measured in all studies. Power for the five tests was displayed when (A) causal variants have unidirectional effect of 0.5, and (B) causal variants have bidirectional effects, i.e. 80% of the causal variants have effect 0.5 and the other 20% have effect −0.5. In the first study, the gene region is associated with the primary trait, and causal variants have an effect of −0.5. The correlation between the primary and secondary traits is 0.6. In the second study, the primary trait is also associated with the gene region, and causal variants have an effect of 0.25. The primary and secondary traits are correlated with coefficient 0.4. In the third study, the gene region is not associated with the primary trait, and the correlation between the primary and secondary traits is −0.2. In each study, a different pool of 50,000 samples was simulated and 2,500 individuals with extreme primary trait were selected and analyzed. P-values for all rare variant tests were obtained based upon 5,000 permutations. The power for analyzing each individual study and meta-analysis was evaluated using 10,000 replicates. (TIF) [file pgen.1003075.s009.tif]
