# Supplementary material for: A Unified Method for Detecting Secondary Trait Associations with Rare Variants: Application to Sequence Data
Source: PLoS Genet. 2012 Nov 15;8(11):e1003075. doi: 10.1371/journal.pgen.1003075 (PMC3499373; doi:10.1371/journal.pgen.1003075)
Supplement: Table S1 — Correlations of phenotypes from the SardiNIA cohort. Eight traits that were analyzed for associations are included, i.e. high density lipoprotein (HDL), low density lipoprotein (LDL), triglyceride (TG), total cholesterol levels (TCL), diastolic blood pressure (DiasBP), systolic blood pressure (SysBP), insulin levels (INSULIN), and body mass index (BMI). Correlations were estimated using 2044 unrelated individuals extracted from the SandiNIA pedigrees. (DOC) [file pgen.1003075.s010.doc]

|  | **HDL** | **LDL** | **TG** | **TCL** | **DiasBP** | **SysBP** | **INSULIN** | **BMI** |
| --- | --- | --- | --- | --- | --- | --- | --- | --- |
| **HDL** | 1.000 | 0.017 | -0.204 | 0.305 | -0.046 | -0.009 | -0.099 | -0.161 |
| **LDL** | 0.017 | 1.000 | 0.002 | 0.877 | 0.203 | 0.145 | -0.003 | 0.137 |
| **TG** | -0.204 | 0.002 | 1.000 | 0.318 | 0.145 | 0.150 | 0.194 | 0.227 |
| **TCL** | 0.305 | 0.877 | 0.318 | 1.000 | 0.216 | 0.182 | 0.038 | 0.149 |
| **DiasBP** | -0.046 | 0.203 | 0.145 | 0.216 | 1.000 | 0.710 | 0.069 | 0.301 |
| **SysBP** | -0.009 | 0.145 | 0.150 | 0.182 | 0.710 | 1.000 | 0.046 | 0.315 |
| **INSULIN** | -0.099 | -0.003 | 0.194 | 0.038 | 0.069 | 0.046 | 1.000 | 0.332 |
| **BMI** | -0.161 | 0.137 | 0.227 | 0.149 | 0.301 | 0.315 | 0.332 | 1.000 |
