# Supplementary material for: A Unified Method for Detecting Secondary Trait Associations with Rare Variants: Application to Sequence Data
Source: PLoS Genet. 2012 Nov 15;8(11):e1003075. doi: 10.1371/journal.pgen.1003075 (PMC3499373; doi:10.1371/journal.pgen.1003075)
Supplement: Table S2 — Analysis of secondary trait associations using standard linear regression. Sample ascertainment mechanisms were ignored in the analysis. Seven secondary traits were analyzed, including total cholesterol levels (TCL), high density lipoprotein (HDL), body mass index (BMI), diastolic blood pressure (DiasBP), systolic blood pressure (SysBP), triglyceride (TG) and insulin levels (INSULIN). Gene-based association analysis was performed using CMC, WSS, KBAC, VT and SKAT. (DOC) [file pgen.1003075.s011.doc]

| **Gene** | **Trait** | **CMC^a,c^** | **WSS^a,c^** | **KBAC^a,c^** | **VT^b,c^** | **SKAT^a,c^** |
| --- | --- | --- | --- | --- | --- | --- |
| *APOB* | TCL | 4.23E-02* | 5.80E-02 | 5.40E-02 | 8.00E-02 | 6.30E-02 |
| *APOB* | HDL | 4.58E-01 | 8.07E-01 | 3.01E-01 | 8.32E-01 | 5.32E-01 |
| *APOB* | BMI | 4.96E-01 | 4.85E-01 | 2.20E-01 | 7.86E-01 | 4.25E-01 |
| *APOB* | DiasBP | 1.05E-01 | 9.50E-02 | 9.40E-02 | 2.15E-01 | 1.65E-01 |
| *APOB* | SysBP | 3.47E-01 | 4.84E-01 | 1.90E-01 | 6.54E-01 | 4.03E-01 |
| *APOB* | TG | 6.37E-02 | 1.06E-01 | 8.10E-02 | 2.15E-01 | 1.93E-01 |
| *APOB* | INSULIN | 3.48E-01 | 4.69E-01 | 2.96E-01 | 5.59E-01 | 2.11E-01 |
| *B3GA4* | TCL | 6.65E-01 | 7.98E-01 | 8.45E-01 | 9.79E-01 | 8.22E-01 |
| *B3GA4* | HDL | 2.52E-01 | 2.90E-01 | 1.48E-01 | 4.08E-01 | 5.60E-02 |
| *B3GA4* | BMI | 7.94E-01 | 8.36E-01 | 9.08E-01 | 9.44E-01 | 9.62E-01 |
| *B3GA4* | DiasBP | 8.54E-01 | 7.86E-01 | 7.56E-01 | 4.26E-01 | 4.89E-01 |
| *B3GA4* | SysBP | 9.03E-01 | 9.30E-01 | 9.11E-01 | 7.42E-01 | 7.90E-01 |
| *B3GA4* | TG | 8.32E-01 | 9.30E-01 | 8.20E-01 | 8.94E-01 | 6.32E-01 |
| *B3GA4* | INSULIN | 9.72E-01 | 9.73E-01 | 9.83E-01 | 9.82E-01 | 9.83E-01 |
| *LDLR* | TCL | 1.06E-01 | 6.10E-02 | 1.00E-01 | 4.80E-02* | 2.51E-01 |
| *LDLR* | HDL | 3.07E-01 | 2.38E-01 | 3.73E-01 | 5.11E-01 | 5.92E-01 |
| *LDLR* | BMI | 3.62E-01 | 4.96E-01 | 5.92E-01 | 7.70E-01 | 9.97E-01 |
| *LDLR* | DiasBP | 6.09E-02 | 9.60E-02 | 1.17E-01 | 1.65E-01 | 7.44E-01 |
| *LDLR* | SysBP | 3.97E-03^#^ | 5.50E-03^#^ | 2.50E-03^#^ | 1.00E-02^#^ | 1.81E-01 |
| *LDLR* | TG | 5.91E-01 | 7.02E-01 | 7.69E-01 | 6.50E-01 | 8.52E-01 |
| *LDLR* | INSULIN | 7.48E-01 | 7.61E-01 | 9.20E-01 | 9.54E-01 | 9.57E-01 |
| *PCSK9* | TCL | 5.72E-01 | 5.19E-01 | 6.82E-01 | 9.35E-01 | 4.00E-01 |
| *PCSK9* | HDL | 4.91E-02* | 7.10E-02 | 8.20E-02 | 9.10E-02 | 2.99E-01 |
| *PCSK9* | BMI | 9.23E-01 | 8.87E-01 | 8.43E-01 | 7.22E-01 | 6.90E-01 |
| *PCSK9* | DiasBP | 9.54E-01 | 9.06E-01 | 6.68E-01 | 9.93E-01 | 4.10E-01 |
| PCSK9 | SysBP | 6.35E-01 | 6.25E-01 | 7.64E-01 | 8.71E-01 | 7.27E-01 |
| PCSK9 | TG | 9.30E-01 | 8.42E-01 | 6.49E-01 | 6.43E-01 | 3.87E-01 |
| PCSK9 | INSULIN | 4.97E-01 | 6.19E-01 | 5.48E-01 | 7.25E-01 | 5.77E-01 |

^a.^For CMC, WSS, KBAC, and SKAT, variants with MAF≤1% were analyzed.

^b.^For VT, variants with MAF≤5% were analyzed.

^c.^Statistical significance for all tests was obtained empirically via 5,000 permutations. Nominally significant p-values are labeled with an asterisk, while the associations that are significant after Bonferroni corrections are labeled with a pound sign.
