# Supplementary material for: A Unified Method for Detecting Secondary Trait Associations with Rare Variants: Application to Sequence Data
Source: PLoS Genet. 2012 Nov 15;8(11):e1003075. doi: 10.1371/journal.pgen.1003075 (PMC3499373; doi:10.1371/journal.pgen.1003075)
Supplement: Text S1 — Extension of Kernel Based Adaptive Cluster to the Analysis of Quantitative Traits. (PDF) [file pgen.1003075.s012.pdf]

The Kernel Based Adaptive Cluster (KBAC) test was originally developed for detecting associations with rare variants in case control studies [1]. In KBAC, the coding function $K^0\left(\vec{X}_i, Y_i^*\right)$ depends on both the multi-site genotype $\vec{X}_i$ and the disease status Y_i^*. Specifically, multi-site genotypes that are more enriched in cases are assigned higher weights, such that potentially causal variants can be distinguished from non-causal variants. The weights are then incorporated into a logistic regression model. Association testing can be performed using score tests and p-values need to be evaluated empirically through permutations. It was shown in Liu and Leal [1], that KBAC can be more powerful than alternative methods in the presence of non-causal variants, or gene interactions.

In order to generalize the KBAC statistics to analyze quantitative traits, it is necessary to extend the kernel weight function $K^0\left(\vec{X}_i, Y_i^*\right)$, which was originally only defined for binary traits. Two binary auxiliary traits are defined, i.e. $Y_{i,H}^* = \delta\left(Y_i > Y^H\right)$, and $Y_{i,L}^* = \delta\left(Y_i < Y^L\right)$, where δ is an indicator function, and $Y_{i,H}^*$ and $Y_{i,L}^*$ are trait cutoffs with $Y_{i,H}^* > Y_{i,L}^*$. When selected samples are used, $Y_{i,H}^*$ and $Y_{i,L}^*$ are set to be equal to the trait thresholds employed for sample ascertainment. When population based random samples are used, as a default, $Y_{i,H}^*$ and $Y_{i,L}^*$ are set to be the 75th and 25th percentiles of the sample quantitative trait values.

In order to test for one-sided hypothesis, e.g. rare causal variants are more enriched in samples with high (or low) trait values, the genotype coding $K\left(\vec{X}_i, Y_i\right) = K^0\left(\vec{X}_i, Y_{i,H}^*\right)$ (or $K\left(\vec{X}_i, Y_i\right) = K^0\left(\vec{X}_i, Y_{i,L}^*\right)$) is used. Score statistics T_H (or T_L) are calculated based upon equation 10, where full quantitative trait is analyzed. If there is no prior information on which extreme

enriches rare causal variants, two-sided hypothesis should be tested and the statistic $T = \max\left\{\left(T^{H}\right)^{2}, \left(T^{L}\right)^{2}\right\}$ is used. Standard permutation algorithms can be used to obtain p-values empirically.

Reference:

1. Liu DJ, Leal SM (2010) A novel adaptive method for the analysis of next-generation sequencing data to detect complex trait associations with rare variants due to gene main effects and interactions. PLoS Genet 6: e1001156.
