# Supplementary material for: A Unified Method for Detecting Secondary Trait Associations with Rare Variants: Application to Sequence Data
Source: PLoS Genet. 2012 Nov 15;8(11):e1003075. doi: 10.1371/journal.pgen.1003075 (PMC3499373; doi:10.1371/journal.pgen.1003075)
Supplement: Text S2 — Biases of Naïve Inferences of Secondary Trait Associations in Selected Samples. (PDF) [file pgen.1003075.s013.pdf]

It was shown previously by simulations, that the naïve analysis of secondary traits can be biased if the ascertainment mechanism is ignored in the analyses [1,2]. In this section, we will quantify the bias rigorously using probability theory.

In order to simplify notation, we assume a model with only one genetic marker and no covariates, i.e.

$$\begin{pmatrix} Y_{1i} \\ Y_{2i} \end{pmatrix} \sim \text{MVN}\left(\begin{pmatrix} \beta_{10} + \tilde{\beta}_{11} X_i \\ \beta_{20} \end{pmatrix}, \begin{bmatrix} \sigma_1^2 & \rho\sigma_1\sigma_2 \\ \rho\sigma_1\sigma_2 & \sigma_2^2 \end{bmatrix} \right)$$

The argument we use is nonetheless general. Under the null hypothesis of no secondary trait association, due to selective sampling, the distribution for the secondary traits follows

$$p\left(Y_{2i} \middle| Y_{1i} \in \left(y^H, \infty\right) \cup \left(-\infty, y^L\right), X_i\right) = \frac{\Pr\left(Y_{1i} \in \left(y^H, \infty\right) \cup \left(-\infty, y^L\right) \middle| Y_{2i}, \vec{X}_i\right) \times p\left(Y_{2i} \middle| X_i\right)}{p\left(Y_{1i} \in \left(y^H, \infty\right) \cup \left(-\infty, y^L\right) \middle| X_i\right)}$$

In fact, it can be seen that if the primary trait is associated with the marker, and the two traits are correlated, then $p\left(Y_{2i} \middle| Y_{1i} \in \left(y^H, \infty\right) \cup \left(-\infty, y^L\right), X_i = 1\right) \neq p\left(Y_{2i} \middle| Y_{1i} \in \left(y^H, \infty\right) \cup \left(-\infty, y^L\right), X_i = 0\right)$.

We plot the likelihoods of $p\left(Y_{2i} \middle| Y_{1i} \in \left(y^H, \infty\right) \cup \left(-\infty, y^L\right), X_i = 1\right)$ and $p\left(Y_{2i} \middle| Y_{1i} \in \left(y^H, \infty\right) \cup \left(-\infty, y^L\right), X_i = 0\right)$ (**Figure S1**), when the primary trait effect is $\tilde{\beta}_{11} = 0.5$ and the residual correlation is $\rho = 0.6$. Clearly the two distributions differ, although they should be identical under the null hypothesis where the secondary trait is not associated to the gene/genetic region.

Using standard probability theory argument, we can calculate

$$E\left(Y_{2i}\middle|Y_{1i}\in\left(y^H,\infty\right)\cup\left(-\infty,y^L\right),X_i\right)=E\left(Y_{2i}\middle|Y_{1i}\in\left(y^H,\infty\right),X_i\right)\times\Pr\left(Y_{1i}\in\left(y^H,\infty\right)\middle|X_i,Y_{1i}\in\left(y^H,\infty\right)\cup\left(-\infty,y^L\right)\right)+$$
$$E\left(Y_{2i}\middle|Y_{1i}\in\left(-\infty,y^L\right),X_i\right)\times\Pr\left(Y_{1i}\in\left(-y^L,\infty\right)\middle|X_i,Y_{1i}\in\left(y^H,\infty\right)\cup\left(-\infty,y^L\right)\right),$$

where $E\left(Y_{2i}\middle|Y_{1i}\in\left(y^H,\infty\right),X_i\right)=\sigma_2\rho\frac{\phi\left(y^H-\tilde{\beta}_{11}X_{1i}/\sigma_1\right)}{\Phi\left(y^H-\tilde{\beta}_{11}X_{1i}/\sigma_1\right)}$ (S2.1)

and

$$E\left(Y_{2i}\middle|Y_{1i}\in\left(-\infty,y^L\right),X_i\right)=\sigma_2\rho\frac{\phi\left(y^L-\tilde{\beta}_{11}X_{1i}/\sigma_1\right)}{1-\Phi\left(y^L-\tilde{\beta}_{11}X_{1i}/\sigma_1\right)} \quad \text{(S2.2)}$$

The functions $\phi(x)$ and $\Phi(x)$ are the density and probability distribution functions for a standard normal random variable. As a result, the bias in the genetic effect of the secondary trait can be analytically quantified. Specifically, when the secondary trait is directly analyzed for associations using a linear regression model without incorporating ascertainment, the bias equals to $E\left(Y_{2i}\middle|Y_{1i}\in\left(y^H,\infty\right)\cup\left(-\infty,y^L\right),X_i=1\right)-E\left(Y_{2i}\middle|Y_{1i}\in\left(y^H,\infty\right)\cup\left(-\infty,y^L\right),X_i=0\right)$.

Similarly, when secondary trait association analysis is performed using individuals with high (or low) extreme primary traits, the bias is given by

$E\left(Y_{2i}\middle|Y_{1i}\in\left(y^H,\infty\right),X_i=1\right)-E\left(Y_{2i}\middle|Y_{1i}\in\left(y^H,\infty\right),X_i=0\right)$ (or

$E\left(Y_{2i}\middle|Y_{1i}\in\left(-\infty,y^L\right),X_i=1\right)-E\left(Y_{2i}\middle|Y_{1i}\in\left(-\infty,y^L\right),X_i=0\right)$).

Another type of naïve analysis is to include an indicator of whether an individual has high or low extreme primary trait value in a linear regression model as covariate i.e.

$Z_i=1\times I\left(Y_i\in\left(y^H,\infty\right)\right)+0\times I\left(Y_i\in\left(-\infty,y^L\right)\right)$, and analyze the secondary trait using the entire

sample. However, this is also incorrect, since conditioning on the indicator Z_i is equivalent to conditioning on $Y_i \in \left(y^H, \infty\right)$ or $Y_i \in \left(-\infty, y^L\right)$, i.e.

$$E\left(Y_{2i} \middle| Z_i = 1, X_i\right) = E\left(Y_{2i} \middle| Y_{1i} \in \left(y^H, \infty\right), X_i\right),\ E\left(Y_{2i} \middle| Z_i = 0, X_i\right) = E\left(Y_{2i} \middle| Y_{1i} \in \left(-\infty, y^L\right), X_i\right)$$

Therefore, naïve methods cannot eliminate the bias in the secondary traits.

It is clear from (S2.1) and (S2.2) that the bias in the effects of the secondary trait is linear with respect to the trait residual correlation ρ. Due to the local properties of inverse Mills ratio $\phi(x)/\Phi(x)$ in the neighborhood of 0, the bias is also approximately linear with respect to the primary trait effect $\tilde{\beta}_{11}$ when $\left|\tilde{\beta}_{11}\right|$ is small. We plotted the biases against different values of the primary trait effect and residual correlation in **Figure S2**, when all individuals are analyzed using a linear regression model without correcting for ascertainment mechanisms.

Reference:

1. Lin DY, Zeng D (2009) Proper analysis of secondary phenotype data in case-control association studies. Genet Epidemiol 33: 256-265.
2. Liu DJ, Leal SM (2011) A flexible likelihood framework for detecting associations with secondary phenotypes in genetic studies using selected samples: application to sequence data. Eur J Hum Genet.
