# Supplementary material for: A Unified Method for Detecting Secondary Trait Associations with Rare Variants: Application to Sequence Data
Source: PLoS Genet. 2012 Nov 15;8(11):e1003075. doi: 10.1371/journal.pgen.1003075 (PMC3499373; doi:10.1371/journal.pgen.1003075)
Supplement: Text S3 — Details for the Null Likelihood Model. (PDF) [file pgen.1003075.s014.pdf]

In order to fully specify the likelihood function in equation 3, we need to calculate $p\left(A_i \middle| Y_{1i}, Y_{2i}\right)$ and $p\left(Y_{1i}, Y_{2i} \middle| \vec{X}_i, \vec{Z}_i\right)$. According the null model, the primary and secondary traits in the general population follow a multivariate normal distribution. Therefore, $p\left(Y_{1i}, Y_{2i} \middle| \vec{X}_i, \vec{Z}_i\right)$ satisfies

$$\begin{pmatrix} Y_{1i} \\ Y_{2i} \end{pmatrix} \sim \text{MVN}\left(\begin{pmatrix} \beta_{10} + \sum_s \beta_{1s} X_i^s + \sum_{j_1} \alpha_{1 j_1} Z_{ij_1} \\ \beta_{20} + \sum_{j_2} \alpha_{2 j_2} Z_{j_2} \end{pmatrix}, \begin{bmatrix} \sigma_1^2 & \rho\sigma_1\sigma_2 \\ \rho\sigma_1\sigma_2 & \sigma_2^2 \end{bmatrix}\right)$$

When selected samples are used, it is assumed that the sampling proportions from each extreme tail are known. For a selective sampling study that collects N^H individuals from the upper extreme (i.e. with primary trait $Y_{1i} \geq y^H$) and N^L individuals from the lower extreme (i.e. with primary trait $Y_{1i} \leq y^L$), the probability $p\left(A_i \middle| Y_{1i}, Y_{2i}\right)$ in the likelihood satisfies

$$p\left(A_i \middle| Y_{1i}, Y_{2i}\right) \propto \begin{cases} N^H \big/ \Pr\left(Y_{1i} \geq y^H\right) & \text{if } Y_{1i} \geq y^H \\ N^L \big/ \Pr\left(Y_{1i} \leq y^H\right) & \text{if } Y_{1i} \leq y^L \end{cases}$$

The model is also applicable to random population based studies, where each individual has equal probability of being sampled. In this case, the sampling probability is proportional to a constant, regardless of the primary and secondary trait values, i.e. $p\left(A_i \middle| Y_{1i}, Y_{2i}\right) \propto C$.

In order to obtain interchangeable residuals under the null hypothesis, the likelihood model needs to be re-parameterized. Specifically, we use $\tau_1 = \sigma_1, \tau_2 = \left(1-\rho^2\right)\sigma_2, \beta_{2r_1} = \sigma_2\rho/\sigma_1$. The Jacobian for the transformation is given by

$$\partial\left(\tau_1,\tau_2,\beta_{2r_1}\right)/\partial\left(\sigma_1,\sigma_2,\rho\right)=\begin{pmatrix} 1 & 0 & 0 \\ 0 & 1-\rho^2 & -2\rho\sigma_2 \\ -\rho\sigma_2/\sigma_1^2 & \rho/\sigma_1 & \sigma_2/\sigma_1 \end{pmatrix}$$. The determinant of

$\partial\left(\tau_1,\tau_2,\beta_{2r_1}\right)/\partial\left(\sigma_1,\sigma_2,\rho\right)$ is always positive and non-zero, i.e.

$\det\left(\partial\left(\tau_1,\tau_2,\beta_{2r_1}\right)/\partial\left(\sigma_1,\sigma_2,\rho\right)\right)=1+\rho^2\,\sigma_2/\sigma_1>0$ Therefore, the transformation is one-to-one and non-degenerate. After the re-parameterization, an equivalent mean model can be fitted,

$$\begin{cases} \mu\left(Y_{1i}\right)=\beta_{10}+\sum_s \beta_{1s}X_s+\sum_{j_1}\alpha_{1j_1}Z_{j_1} \\ \mu\left(Y_{2i}\right)=\beta_{20}+\beta_{2r_1}\left(Y_{1i}-\mu\left(Y_{1i}\right)\right)+\sum_{j_2}\alpha_{2j_2}Z_{j_2} \end{cases}.$$

The residuals $e_{1i}=Y_{1i}-\mu\left(Y_{1i}\right)$ and $e_{2i}=Y_{2i}-\mu\left(Y_{2i}\right)$ from the model are interchangeable under the null hypothesis.
