# Supplementary material for: A Unified Method for Detecting Secondary Trait Associations with Rare Variants: Application to Sequence Data
Source: PLoS Genet. 2012 Nov 15;8(11):e1003075. doi: 10.1371/journal.pgen.1003075 (PMC3499373; doi:10.1371/journal.pgen.1003075)
Supplement: Text S4 — Practical Issues for Inferences under the Ascertainment Corrected Likelihood. (PDF) [file pgen.1003075.s015.pdf]

Under the ascertainment corrected likelihood model, the primary and secondary traits are jointly modeled, i.e. $p\left(Y_{1i}, Y_{2i} \middle| A_i, \vec{X}_i\right)$. As the samples are ascertained based upon the primary trait, the sampling probability satisfies

$$
\begin{aligned}
p\left(Y_{2i} \middle| A_i, Y_{1i}, \vec{X}_i\right) &= p\left(Y_{2i} \middle| Y_{1i}, \vec{X}_i\right) \\
&\sim \mathrm{N}\left(\mu_2 + \rho\sigma_2/\sigma_1\left(Y_{1i} - \mu\left(Y_{1i}\right)\right), \sigma_2^2\left(1-\rho^2\right)\right)
\end{aligned}
$$

In order to perform unbiased inferences for the secondary traits, it is essential to correctly estimate the conditional mean value for the primary trait $\mu\left(Y_{1i}\right)$. Since the primary trait effects of different variants may differ, the secondary trait distribution conditional on each multi-site genotype coding $K\left(\vec{X}_i\right)$ and the primary trait Y_{1i} can also be different. Therefore, the primary trait effect for each variant should ideally be estimated individually. However, for nucleotide sites where only a few copies of variant alleles (<5) are observed, estimating their effects can be numerically unstable. In our implementation, as a default, we collapse nucleotide sites with <5 copies of variant alleles. The results of our simulation suggest that the maximum likelihood estimation procedure under this collapsing scheme is numerically stable and the score statistics have controlled type-I errors in all scenarios. We also evaluated STAR using a few other choices of the variant count thresholds, and the results remain similar (data not shown).
