# Supplementary material for: A Unified Method for Detecting Secondary Trait Associations with Rare Variants: Application to Sequence Data
Source: PLoS Genet. 2012 Nov 15;8(11):e1003075. doi: 10.1371/journal.pgen.1003075 (PMC3499373; doi:10.1371/journal.pgen.1003075)
Supplement: Text S5 — Constructing Variance Component Score Tests from Ascertainment Corrected Likelihood. (PDF) [file pgen.1003075.s016.pdf]

In a variance component model, the genetic effects for rare variants from each nucleotide site are modeled as random effects with mean 0 and variance η^2, i.e. $dP\left(\vec{\beta}_2;0,\eta^2\right)$. According to the work by Lin [1], the variance component score statistics can be calculated based upon the marginal likelihood,

$$p\left(\vec{Y}_1,\vec{Y}_2\middle|A_i,\vec{X}_i;\eta,\tau_1,\tau_2,\mu(Y_{i1}),\mu(Y_{i2})\right)=\int\prod_i p\left(\vec{Y}_{i1},\vec{Y}_{i2}\middle|A_i,\vec{X}_i;\vec{\beta}_2,\tau_1,\tau_2,\mu(Y_{i1}),\mu(Y_{i2})\right)dP\left(\vec{\beta}_2;0,\eta^2\right)\text{(S5.1)}$$

However, the calculation using (S5.1) is not straightforward. Instead, the following theorem can be applied:

Theorem: The variance component score statistics calculated using (S5.1) can be equivalently constructed based upon the conditional likelihood, i.e.

$$p\left(\vec{Y}_2\middle|\vec{Y}_1,\vec{X}_i;\eta,\tau_1,\tau_2,\mu(Y_{i1}),\mu(Y_{i2})\right)=\int\prod_i p\left(Y_{i2}\middle|Y_{i1},\vec{X}_i;\vec{\beta}_2,\tau_1,\tau_2,\mu(Y_{i1}),\mu(Y_{i2})\right)dP\left(\vec{\beta}_2;0,\eta^2\right)\ \text{(S5.2)}$$

Therefore the variance component score statistic has the form

$$S=\left(\vec{Y}_2-\hat{\mu}\left(\vec{Y}_2\right)\right)^T Ker\left(\vec{X},\vec{X}\right)\left(\vec{Y}_2-\hat{\mu}\left(\vec{Y}_2\right)\right)$$

where $Ker\left(\vec{X}_i,\vec{X}_j\right)$ is the kernel function for comparing two multi-site genotypes, and $\hat{\mu}\left(\vec{Y}_1\right)$, $\hat{\mu}\left(\vec{Y}_2\right)$ are the estimated mean primary and secondary trait values under the null model.

Proof: In order to prove the theorem, we first factorize the probability $p\left(Y_{i1},Y_{i2}\middle|A_i,\vec{X}_i;\vec{\beta}_2,\tau_1,\tau_2,\mu(Y_{i1}),\mu(Y_{i2})\right)$, i.e.

$$\begin{aligned}p\left(Y_{i1},Y_{i2}\middle|A_i,\vec{X}_i;\vec{\beta}_2,\mu(Y_{i1}),\mu(Y_{i2})\right)&=p\left(Y_{i2}\middle|A_i,Y_{i1},\vec{X}_i;\vec{\beta}_2,\tau_1,\tau_2,\mu(Y_{i1}),\mu(Y_{i2})\right)\\&\quad\times p\left(Y_{i1}\middle|A_i,\vec{X}_i;\tau_1,\tau_2,\mu(Y_{i1}),\mu(Y_{i2})\right)\end{aligned}.$$

The term $p\left(Y_{i1}\middle|A_i,\vec{X}_i;\tau_1,\tau_2,\mu(Y_{i1}),\mu(Y_{i2})\right)$ does not contain the parameters $\vec{\beta}_2$, and therefore can be taken out of the integral in (S5.1), i.e.

$$p\left(\vec{Y}_1,\vec{Y}_2\middle|A_i,\vec{X}_i;\tau,\mu(Y_{i1}),\mu(Y_{i2})\right)=\int\prod_i p\left(Y_{i2}\middle|Y_{i1},A_i,\vec{X}_i;\vec{\beta}_2,\tau_1,\tau_2,\mu(Y_{i1}),\mu(Y_{i2})\right)dP\left(\vec{\beta}_2;0,\eta^2\right)$$
$$\times p\left(Y_{i1}\middle|A_i,\vec{X}_i;\tau_1,\tau_2,\mu(Y_{i1}),\mu(Y_{i2})\right)$$

If the sample ascertainment is only based upon the primary trait, using the same argument as in equations (8) and (9), we can obtain

$$p\left(Y_{i2}\middle|Y_{i1},A_i,\vec{X}_i;\vec{\beta}_2,\tau_1,\tau_2,\mu(Y_{i1}),\mu(Y_{i2})\right)=p\left(Y_{i2}\middle|Y_{i1},\vec{X}_i;\vec{\beta}_2,\tau_1,\tau_2,\mu(Y_{i1}),\mu(Y_{i2})\right) \text{ and}$$

$$p\left(Y_{i2}\middle|Y_{i1},A_i,\vec{X}_i;\tau_1,\tau_2,\mu(Y_{i1}),\mu(Y_{i2})\right)=p\left(Y_{i2}\middle|Y_{i1},\vec{X}_i;\tau_1,\tau_2,\mu(Y_{i1}),\mu(Y_{i2})\right)$$

Therefore, the variance component score statistics can be equivalently calculated from

$$p\left(\vec{Y}_2\middle|\vec{Y}_1,\vec{X}_i;\tau_1,\tau_2,\vec{\beta}_2,\mu(Y_{i1}),\mu(Y_{i2})\right)=\int\prod_i p\left(Y_{i2}\middle|Y_{i1},\vec{X}_i;\tau_1,\tau_2,\vec{\beta}_2,\mu(Y_{i1}),\mu(Y_{i2})\right)dP\left(\vec{\beta}_2;0,\eta^2\right).$$

Given that $\hat{\mu}(Y_{i1}),\hat{\mu}(Y_{i2})$ are consistent estimators under the null hypothesis, by Slutsky's theorem [2], $Y_{i2}-\hat{\mu}(Y_{i2})$ is approximately normally distributed conditional on $Y_{i1},\vec{X}_i$, i.e. $Y_{i2}-\hat{\mu}(Y_{i2})\sim \mathrm{N}\left(0,\hat{\tau}_2^2\right)$ Therefore, according to Wu et al [3], the SKAT statistic is given by

$$S=\left(\vec{Y}_2-\hat{\mu}\left(\vec{Y}_2\right)\right)^T Ker\left(\vec{X},\vec{X}\right)\left(\vec{Y}_2-\hat{\mu}\left(\vec{Y}_2\right)\right).$$

The SKAT statistic follows a mixture chi-square distribution with the mixture proportions being the eigen values for $Ker\left(\vec{X},\vec{X}\right)\widehat{\mathrm{var}}\left(\vec{Y}_2-\hat{\mu}\left(\vec{Y}_2\right)\right)$.

Reference:

1. Lin X (1997) Variance component testing in generalised linear models with random effects. Biometrika 84: 309-326.
2. Resnick SI (1999) A probability path. Boston: Birkhauser. 453 p. p.
3. Wu MC, Lee S, Cai T, Li Y, Boehnke M, et al. (2011) Rare-variant association testing for sequencing data with the sequence kernel association test. Am J Hum Genet 89: 82-93.
